# Adaptive laboratory evolution of *Corynebacterium glutamicum* towards higher growth rates on glucose minimal medium

**DOI:** 10.1038/s41598-017-17014-9

**Published:** 2017-12-01

**Authors:** Eugen Pfeifer, Cornelia Gätgens, Tino Polen, Julia Frunzke

**Affiliations:** Institute of Bio- und Geosciences, IBG-1: Biotechnology, Forschungszentrum Jülich GmbH, 52425 Jülich, Germany

## Abstract

In this work, we performed a comparative adaptive laboratory evolution experiment of the important biotechnological platform strain *Corynebacterium glutamicum* ATCC 13032 and its prophage-free variant MB001 towards improved growth rates on glucose minimal medium. Both strains displayed a comparable adaptation behavior and no significant differences in genomic rearrangements and mutation frequencies. Remarkably, a significant fitness leap by about 20% was observed for both strains already after 100 generations. Isolated top clones (UBw and UBm) showed an about 26% increased growth rate on glucose minimal medium. Genome sequencing of evolved clones and populations resulted in the identification of key mutations in *pyk* (pyruvate kinase), *fruK* (1-phosphofructokinase) and *corA* encoding a Mg^2+^ importer. The reintegration of selected *pyk* and *fruK* mutations resulted in an increased glucose consumption rate and *ptsG* expression causative for the accelerated growth on glucose minimal medium, whereas *corA* mutations improved growth under Mg^2+^ limiting conditions. Overall, this study resulted in the identification of causative key mutations improving the growth of *C. glutamicum* on glucose. These identified mutational hot spots as well as the two evolved top strains, UBw and UBm, represent promising targets for future metabolic engineering approaches.

## Introduction

In recent years, adaptive laboratory evolution experiments (ALE) in combination with next-generation sequencing (NGS) became a key approach to study microbial adaptation in fundamental as well as in applied research^1–4^. Especially in the field of metabolic engineering, researchers took advantage of the fast adaptation of microbes towards changing environments^4,5^. Here, ALE approaches represent a powerful complementary strategy to rational strain engineering to improve growth, product tolerance or stress resistance^2,4–9^. Now, even synthetic ALE scenarios are emerging where synthetic regulatory circuits are implemented to impose an artificial selection pressure on a particular phenotypic trait (e.g. small molecule production)^10–12^. In the course of ALE experiments, spontaneous mutations and genomic rearrangements bearing a fitness advantage under the particular selection pressure establish within the population. However, the overall setup of the ALE experiment has a remarkable impact on the outcome. Important parameters include for example the mode of propagation (batch *versus* continuous culture), the passage size and the growth phase under which cells are transferred to the fresh medium^13–15^. A number of recent reviews nicely summarize current efforts in this scientific field^4,11,15^.

Mutation and selection are the key drivers in ALE experiments. However, the activity of specialized mobile elements^16^, in particular, transposable phages, genomic islands and cryptic prophages may have a considerable influence on the stability of bacterial genomes by causing genomic rearrangements e.g. integrations, deletions, disruptions or inversions^16–18^. Consequently, these elements represent prime candidates for removal in several recent genome reduction projects aiming at the construction of stable and predictable chassis strains^19,20^. However, the impact of suchlike genomic modifications on the long-term genomic stability, mutation frequency and evolvability of the particular strain is usually not characterized.

In this study, we focused on *Corynebacterium glutamicum* representing one of the most important industrial platform organism used for the production of L-glutamate and L-lysine (about 3.1 million and 2.2 million tons per year, respectively)^21,22^ and various further value-added products^23–26^. Although laboratory evolution experiments with *C. glutamicum* proved to achieve promising results^6,10,27^ investigations on long-term scales have not yet been performed. The genome of this Gram-positive soil bacterium contains three cryptic prophage elements (CGP1–3) of which the largest element, CGP3, is still inducible leading to death of the affected cell^28,29^. In a recent study, all three cryptic elements have been removed from the genome of *C. glutamicum* ATCC13032 resulting in strain MB001 with a genome reduced by 6%^19^. This prophage-free variant prevailed to be a stable strain for metabolic engineering as reflected by several recent studies^19,30–33^.

In this work, we compared *C. glutamicum* ATCC 13032 wild type strain and its prophage-free variant MB001 in a long-term evolution experiment on glucose minimal medium. For both strains, a fast adaptation resulting in about ~20% increased growth rates was observed within the first ~100 generations. Genome sequencing of population samples as well as selected isolates revealed frequently occurring key mutations in *pyk* (pyruvate kinase) and *fruK* genes (*pfkB*, 1-phosphofructokinase) leading to increased glucose uptake and growth rates when reintroduced into the wild type background. Overall, our results revealed no significant differences between the two strains in terms of mutation frequency and genomic stability and even emphasized a positive trend of MB001 to evolve to higher growth rates under the chosen conditions.

## Results

### Competitive growth of the prophage-free *C. glutamicum* strain MB001

**Figure 1:**
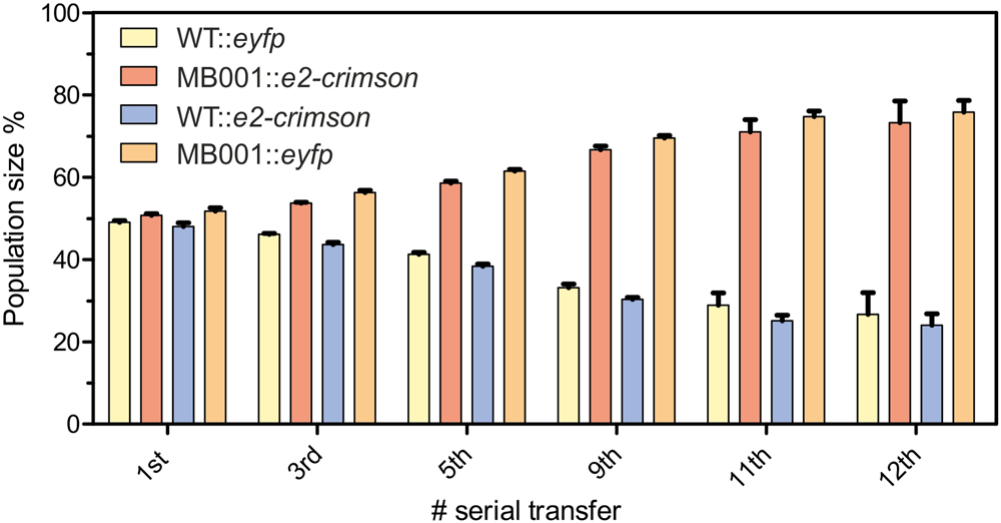
Competitive growth experiment of *C. glutamicum* ATCC 13032 and MB001. *C. glutamicum* ATCC 13032 and MB001 strains were both labelled *vice versa* with a genomically encoded yellow or far red fluorescent protein (eYFP and E2-crimson)^19^. The resulting strains were compared *vice versa* in a competitive growth study on CGXII minimal medium containing 2% (w/v) glucose. Cells were serially transferred to fresh medium after 24 h. Production of the fluorescent proteins was induced by adding IPTG in a final concentration of 0.5 mM six hours before samples for flow cytometry analysis were taken. Strains from the co-cultivation experiment were discriminated by flow cytometry determining the fraction of eYFP and E2-Crimson positive cells.

In previous studies, we reported on the construction of a prophage-free variant of *C. glutamicum* strain ATCC 13032 named MB001 displaying several positive features for metabolic engineering^19^. Interestingly, these studies also revealed a slightly but not significantly increased growth rate in comparison to the wild type strain. To test for a competitive growth advantage of MB001, fluorescent reporter genes were integrated into the genome of the wild type strain ATCC 13032 and MB001^19^. With the resulting strains (mixed 1:1), a competitive growth experiment was conducted in glucose minimal medium. The composition of the population was analyzed by flow cytometry for twelve serial transfers. Remarkably, already after three serial transfers a significant competitive advantage for strain MB001 (for both reporter strains, *vice versa*) was observed (Fig. 1). This finding supports a slightly increased growth rate of the prophage-free MB001 strain compared to the wild type. However, we wondered whether the lack of all prophage regions (~6% of the genome) would somehow affect the genomic stability or evolvability of MB001 and addressed this question by an evolution experiment presented in the following.

### Adaptive evolution experiment on glucose minimal medium

**Figure 2:**
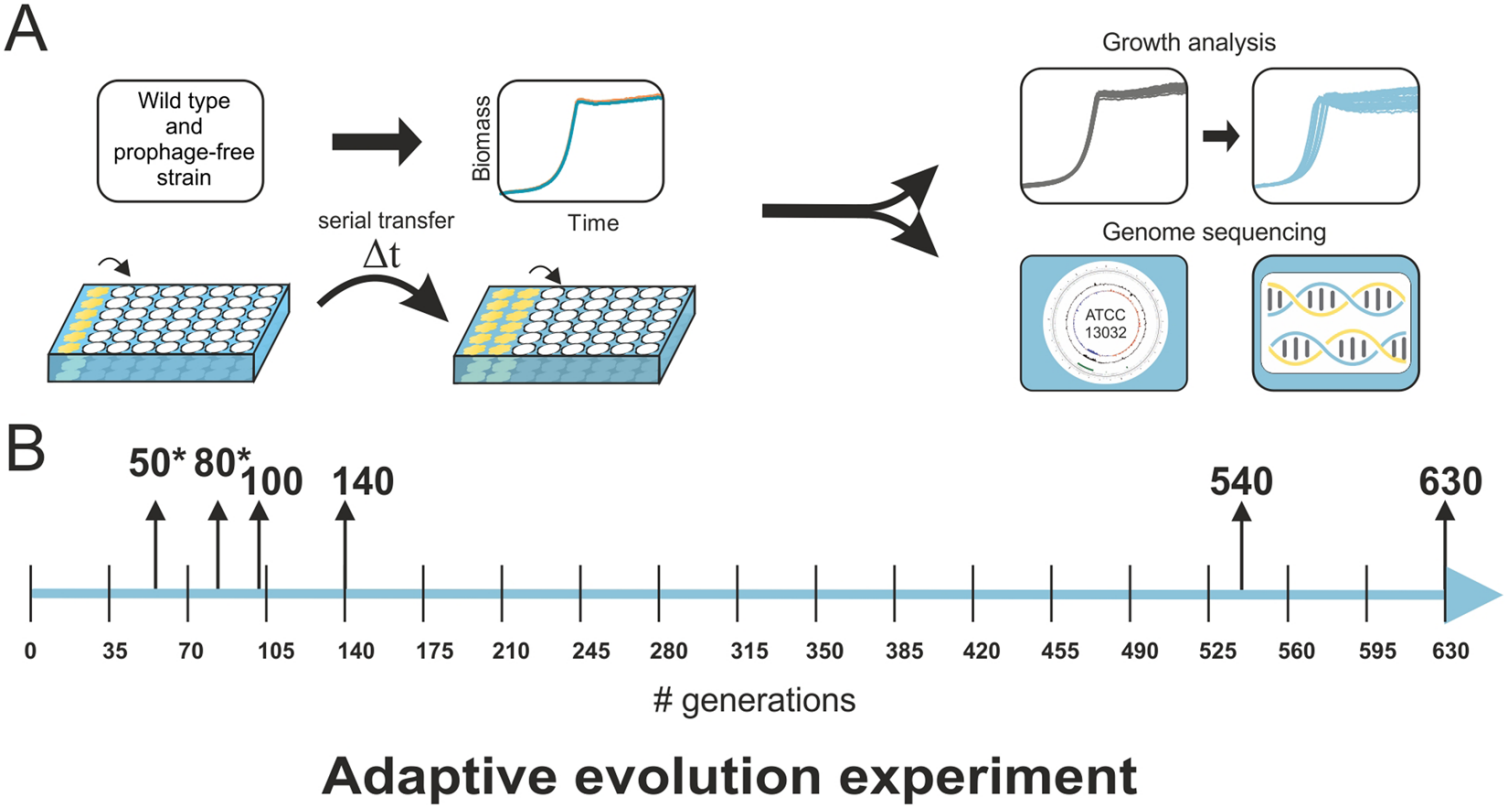
Adaptive evolution experiment. (**A**) *C. glutamicum* ATCC 13032 and its prophage-free variant MB001 were adaptively evolved towards increased growth rates on glucose minimal medium (CGXII medium with 2% (w/v) glucose). Overall, six independent cell lines for each strain were evolved by repetitive batch cultivation using 48-well plates incubated at 30 °C (experimental details are given in the material and methods section). (**B**) Time line of the ALE experiment. Highlighted are the samples that were further analyzed by genome sequencing. Samples of the second reproduction of the experiment are marked with an asterisk.

In the evolution experiment, the robustness and adaptive potential of *C. glutamicum* ATCC 13032 and its prophage-free variant MB001 were compared. Prior to the ALE experiment, both strains were genomically barcoded by a short unique DNA sequence to trace the particular strains throughout the experiment and detect contaminations. Cells were grown in repetitive batch cultures in glucose minimal medium (CGXII with 2% (w/v) glucose) and serially transferred (90 times) from the stationary phase into fresh medium (Fig. 2). For each strain, six independent cells lines were adaptively evolved in parallel for about ~630 generations. Frozen glycerol stocks were prepared throughout the experiment to allow further analysis and/or re-inoculations (for details see material and methods).

**Figure 3:**
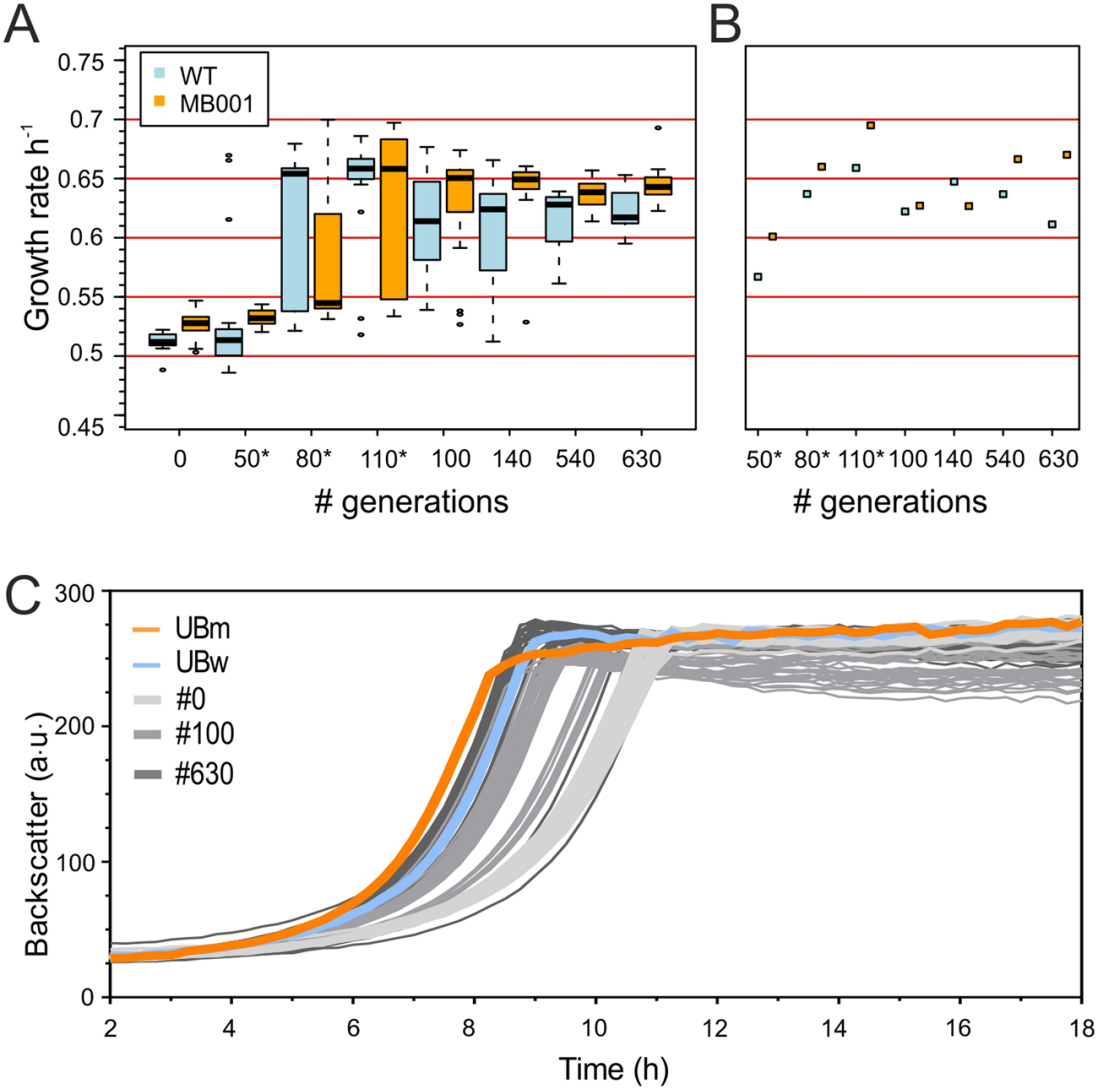
Fitness improvement of evolved strains. Growth rates of 24 clones isolated from each strain at the indicated time point (**A**) as well as for the population mixture (**B**) are shown (for the results of the second cell line, see Figure S1B). To highlight outliers, whiskers of the boxplot represent values within 1.5 IQR. Samples marked with an asterisk are derived from the repetition of the ALE experiment. (**C**) The growth of the top isolated strains UBm (MB001 derivative) and UBw (based on ATCC 13032) is compared to clones isolated at the beginning of the experiment (#0), after 100 and after 630 generations.

The overall fitness in terms of growth rate was analyzed at indicated time points at the population level as well as for 24 isolates for each strain (Fig. 3). Remarkable, a significant increase in fitness was observed for all six cell lines after approx. 100 generations (Fig. 3). The box plot shown in Fig. 3A illustrates the high diversity of the populations narrowing again after about 140 generations coinciding with an overall increase of competitive fitness. In this initial phase of the experiment ATCC 13032 exhibited an increase in growth rate from 0.51 ± 0.01 h^−1^ to 0.62 ± 0.04 h^−1^, whereas MB001 strains adaptively evolved from 0.53 ± 0.01 h^−1^ to 0.65 ± 0.03 h^−1^. This adaptation step was reproduced in a second ALE experiment, where a significant fraction of isolated clones exhibited a leap in their fitness after about 80 generations. Differences in the final OD_600_ values of stationary cells were not observed (Figure S1A) and both evolved strains behaved comparable regarding their sensitivity heat stress and UV (Figure S2). However, the evolved cell lines showed a significantly improved tolerance of osmotic stress (1 M NaCl) in comparison to the parental strains reflecting the adaptation to the high salt conditions in the CGXII medium (Figures S2 and S3).

Finally, we present the two isolated top strains of this ALE experiment named UBw (originating from ATCC 13032, µ = 0.64 ± 0.01 h^−1^) and UBm (MB001, µ = 0.67 ± 0.01 h^−1^). These strains featured the highest growth rate (UB = Usain Bolt) observed for the isolated clones and were therefore analyzed in more detail in the following experiments. Improved growth behavior was also demonstrated in standard shaking flask experiments, where both strains revealed an average increase in growth rate of more than 20% in comparison to their parental strain (Figure S4).

### Adaptation on the genomic level

A central goal of the ALE experiment is the identification of key mutations causing an improvement in fitness under the respective selective conditions. For this purpose, genome sequencing of two cell lines (out of six) of the two strains was performed at the first and last two time points (after 100, 140, 540 and 630 generations). Mapping of the read data to the published genome sequences^19,34^ revealed several transposon insertions and 70 potentially causative SNPs (Tables S6 & S7, Figure S5)). Additionally, five transposon insertions were observed for the wild type as well as for the prophage-free strain (Table S7). As expected the number of SNPs increased over time for all investigated cell lines (Figure S6), but no significant differences, neither in the mutation frequency nor in transposon activity, were observed between ATCC 13032 and MB001 (Figure S6, Table S7).

**Figure 4:**
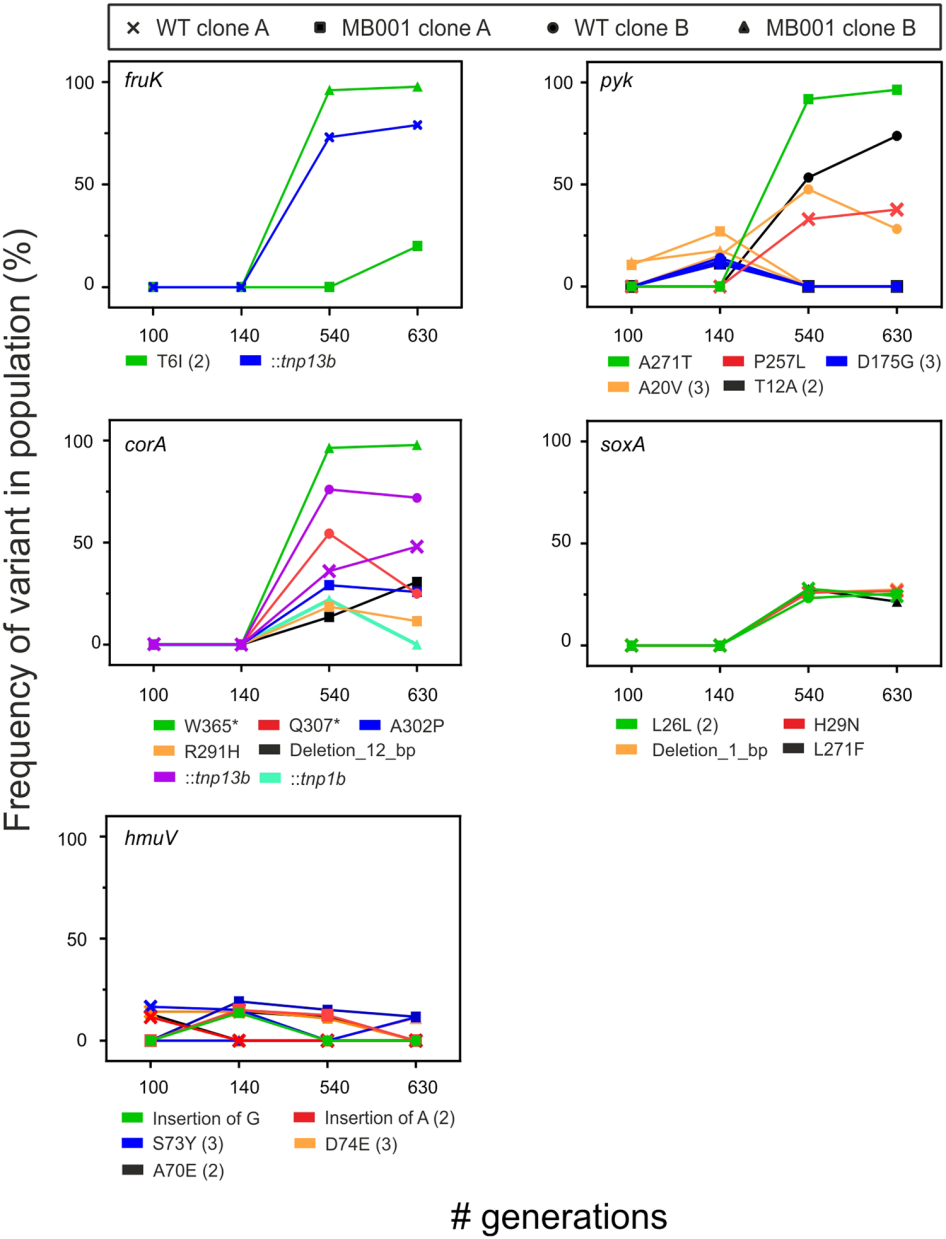
Frequencies of mutations in selected genes at different time points. Mutations affecting the same gene are grouped in one graph. The number in brackets behind the mutation indicates how often a particular mutation was observed in independent cell lines. Data of two wild type (cross, circle) and prophage-free cell lines (square, triangle) are shown for each investigated time point (approx. 100, 140, 540 and 630 generations) of the main ALE experiment. Frequency of mutations was defined as the count of the mutation divided by the coverage measured at this position.

To identify causative key mutations, the SNPs were clustered with respect to the time of their appearance in the ALE experiment and their frequency within a particular gene or genomic region (Tables 1 and 2). Considering that all six initial cell lines displayed improved fitness after ~100 generations on glucose minimal media (Fig. 3), we were particularly interested in mutations appearing in this early stage of the ALE experiment. Remarkably, the *pyk* gene, encoding a pyruvate kinase, was one of the most frequently affected genes with five different mutations (Table 1, Fig. 4). Three *pyk* mutations (D175G, A20V and T12A) were observed independently in different strains at different time points. In particular, these *pyk* mutations already appeared in an early stage of the experiment (after 100 & 140 generations) in low frequencies and displayed a competitive dynamic progression within the population (Fig. 4, Table S4). However, significant differences in the persistence of *pyk* mutations were observed: Whereas, for example, mutation A271T, which is also present in the UBm strain (Table 2), increased in frequency from 0 to almost 100%), mutation A20V (orange circles) and T12A (black circles) were almost equal after 540 generations but a shift towards T12A was observable after 630 generations (Fig. 4, Table S4).

**Table 1:** Key mutations identified in the ALE experiment.

Locus	Gene	#Mut.	Annotation
cg0080	*corA*	7	putative CorA-like Mg^2+^/Co^2+^ transporter protein, MIT-family
cg2291	*pyk*	5	pyruvate kinase (EC:2.7.1.40)
cg0469	*hmuV*	5	hemin transport system, ATP-binding protein
cg1781_cg1783*	*soxA*	4	sarcosine oxidase, cg1781 encodes the C-terminal fragment, cg1783 encodes the N-terminal fragment
cg2807, cg2600	*tnp*	4	transposase, putative pseudogene
cg0418	—	3	putative aminotransferase, involved in cell wall biosynthesis
cg3213	—	3	putative secreted protein
cgtRNA_3558	Leu tRNA	3	cgtRNA_3558, Leu tRNA
cg2119	*fruK* (*pfkB*)	3	1-phosphofructokinase (EC:2.7.1.56)
cg2136	*gluA*	2	glutamate uptake system, ABC-type, ATP-binding protein
cg1419-cg1420	*gatB* (cg1420)	2	putative Na^+^-dependent transporter

Mutations (for complete list, see Figure S4) were clustered according to their frequencies in the respective genes. For a complete list of all mutations, see Table S6.

^*^cg1782 encodes a transposase.

**Table 2:** Mutations identified in the evolved strains UBw and UBm.

Gene/Locus	Annotation	Mutation	UBw Freq^##^	Ubm Freq^##^
*corA*, cg0080	Putative CorA-like Mg^2+^/Co^2+^ transporter protein, MIT-family	Insertion of *tnp13b* (cg1782)	0.78^##^	0
*corA*, cg0080	Putative CorA-like Mg^2+^/Co^2+^ transporter protein, MIT-family	Deletion 12 bp, CGTCGACGATGG, position 593 to 604^#^	0	68/69
*hmuV*, cg0469	hemin transport system, ATP-binding protein	Exchange S73Y	5/46	0
*whmD*, cg0850	*whmD* homolog	G to A mutation, 181 bp upstream^#^	0	166/166
-, cg0934	hypothetical protein, conserved	Deletion 1 bp, T at position 1456^#^	0	151/160
Intergenic, cg1696 and *aspA*, cg1697	cg1696, permease of the major facilitator superfamily and *aspA*, aspartate ammonia-lyase (aspartase) (EC 4.3.1.1)	Insertion of *tnp13b* (cg1782) in IGR	0	0.60
*psp1*, cg2069	cg2069, *psp1*, putative secreted protein, CGP3 region	Exchange V123L	125/125	0
*soxA*, cg1781	soxA, sarcosine oxidase- C-terminal fragment	Exchange L26L, codon exchange TTA to CTA	83/207	
*fruK*, cg2119	1-phosphofructokinase (EC:2.7.1.56)	Exchange R71L	0	168/168
*pyk*, cg2291	pyruvate kinase (EC:2.7.1.40)	Exchange A20V	60/60	0
*pyk*, cg2291	pyruvate kinase (EC:2.7.1.40)	Exchange A271T	0	171/171
-, cg2380	putative membrane protein	Deletion 1 bp, C, position 270^#^	121/124	0
-, cg2380	putative membrane protein	Stop Q93*	0	159/159
*psp5*, cg3197	putative secreted protein	S270S	142/142	0
cg3226	lactate permease	Insertion of *tpn5a* (cg0824)	0.91^##^	0

^**#**^Position 1 is the first bp of the start codon from the respective gene.

^##^Frequencies of SNVs (or MNVs) are given by the count/coverage ratio and transposon insertions are described by the ratio to the variant sequence.

Further targets frequently hit by mutations were *corA*, encoding a CorA-like Mg^2+^/Co^2+^ transporter, *hmuV*, encoding a component of a hemin transport system, *soxA* (sarcoside oxidase and *fruK* (*pfkB*) encoding a phosphofructokinase. Especially mutations in *hmuV* were observed many times, but displayed no competitive progression in the population. The frequencies of mutations found in *hmuV* and *soxA* were low (<30%) in comparison to other mutations.

However, analysis of the sequencing data revealed the phosphofructokinase gene *fruK* as another important key target. Within this experiment, *fruK* was hit by two mutations (T6I and R71L) and by an insertion of a transposon (Fig. 4, Table S5). It is noteworthy that one of the two *fruK* SNPs (T6I) occurred independently at different time points in the two prophage-free cell lines whereas mutation R71L was found only in strain UBm showing the highest growth rate of all isolated clones. In the repetition of the initial phase of the ALE experiment *fruK* was hit by a transposon insertion at a time point where a significant jump in fitness was occurring (Table S5). Besides *pyk* and *fruK*, also different SNPs as well as transposon insertions were found in the *corA* gene. These were, however, only observed at later stages in the ALE experiment suggesting epistatic interactions of *corA* mutations supporting the evolution towards higher growth rates on glucose (Fig. 3, Tables S4 and S7).

### Impact of *pyk*, *fruK* and *corA* mutations

In the following, we studied the impact of selected *pyk*, *fruK* and *corA* mutations on *C. glutamicum* fitness by reintroduction into the ATCC 13032 (WT) strain background (Table 3). Due to low frequencies of *soxA* and *hmuV* mutations (<30%, Fig. 4, Table S4) we assumed them not to be of central importance for the improved growth on glucose. The *pyk* mutations A271T, A20V and T12A were selected, since the latter two differed in their competitive behavior (A20V and T12, Fig. 4) and A271T was identified as the only *pyk* mutation in UBm. Besides the two *fruK* mutations T6I and R71L, the non-sense mutation Q307* in *corA* and the impact of a 12-bp deletion (near the C-terminus) were also examined in the parental strain background.

**Table 3:** Comparative analysis of evolved strains and strains carrying selected key mutations.

Strain	Growth rate (h^−1^) (glucose)	Growth rate fold change (%) (glucose)	Glucose consumption rate (nmol·min^−1^·mg^−1^)	ptsG expression* (a.u.) (glucose)	PK activity (mU·(mg protein)^−1^)	Growth on fructose**	Growth on gluconate**
ATCC 13032	0.52 ± 0.01	0.0 ± 1.9	89.3 ± 1.1	1 ± 0.01	299.4 ± 42.4	+/−	+/−
MB001	0.52 ± 0.01	0.0 ± 1.9	92.8 ± 1.6	1.01 ± 0.01	259.0 ± 23.6	+/−	+/−
UBw	0.64 ± 0.01	23.1 ± 2.1	99.4 ± 9.4	1.51 ± 0.02	85.0 ± 50.7	+/−	−−
UBm	0.67 ± 0.01	28.8 ± 1.9	125.6 ± 2.5	1.52 ± 0.01	266.7 ± 44.6	+ +	+
WT_*pyk*_T12A	0.62 ± 0.01	19.2 ± 1.9	90.76 ± 3.8	1.35 ± 0.01	18.2 ± 15.0	+/−	−−
WT_*pyk* A20V	0.64 ± 0.01	23.1 ± 1.9	N.D.	1.29 ± 0.01	69.3 ± 17.4	+/−	−−
WT_*pyk* A271T	0.57 ± 0.02	9.6 ± 3.8	101.4 ± 8.7	1.24 ± 0.01	337.1 ± 53.1	+	+/−
WT_*fruK* R71L	0.56 ± 0.00	7.7 ± 0.6	106.5 ± 9.8	1.16 ± 0.00	N.D.	++	+
WT_*fruK_*T6I	0.60 ± 0.01	15.4 ± 1.9	N.D.	1.26 ± 0.01	N.D.	−	+
WT_*corA_* Q307*	0.53 ± 0.01	1.9 ± 1.9	N.D.	0.99 ± 0.01	N.D.	+/−	+/−
WT_*corA_*Δ12 bp	0.44 ± 0.01	−15.4 ± 1.9	N.D.	1.01 ± 0.02	N.D.	−	−

N.D.: not determined.

*Normalized to wild type level (Figure S6B).

**Evaluation is based on the growth analysis shown in Figure S7, the wild type strain served as reference ++ strongly increased, + increase, +/− no difference,− impeded, −− strongly impaired.

**Figure 5:**
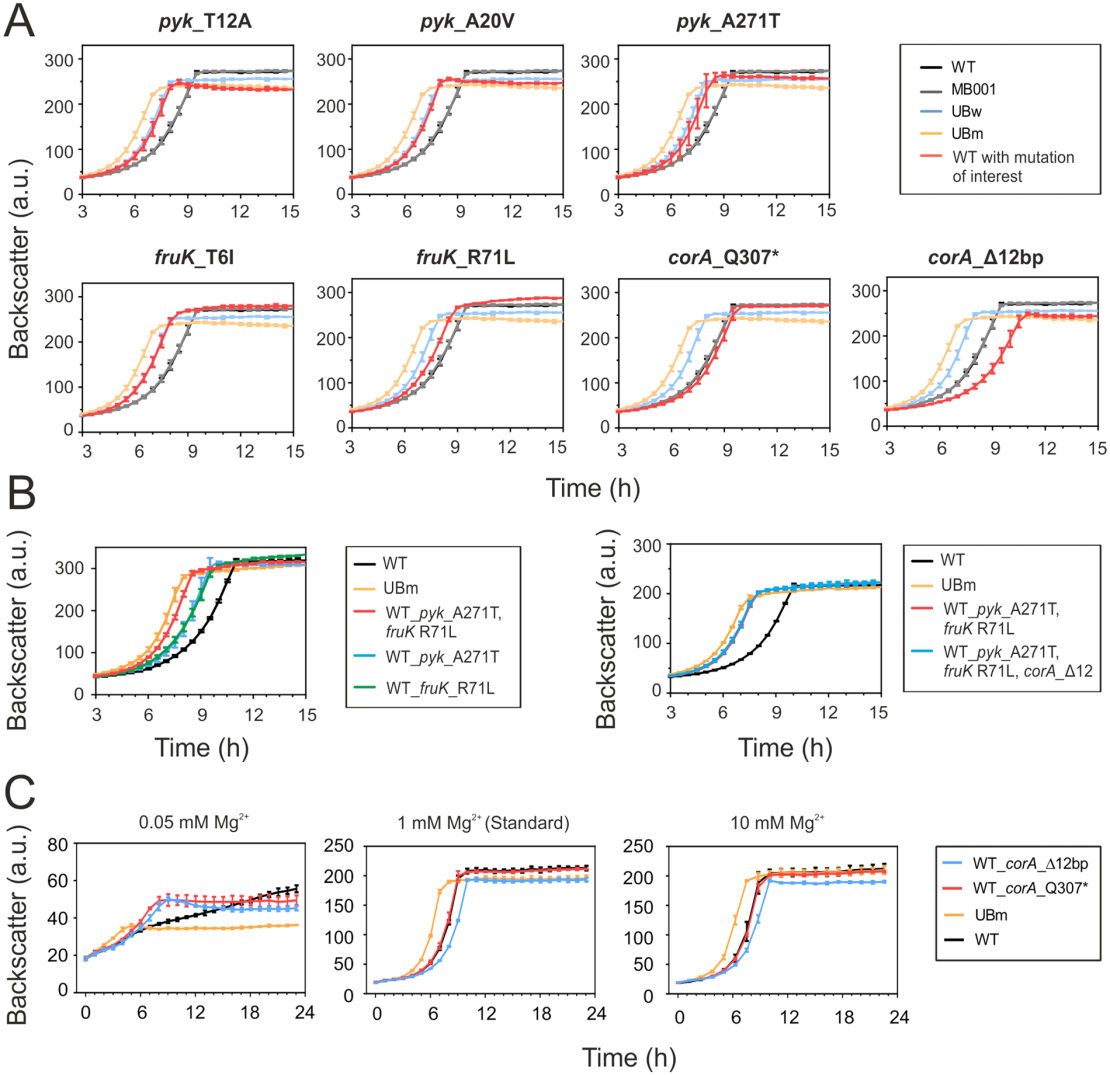
Impact of key mutations on the fitness in glucose minimal medium (**A**) Key mutations identified in *pyk*, *fruK* and *corA* (see Table 2) were introduced into the parental wild type strain *C. glutamicum* ATCC 13032 and investigated regarding their impact on growth in CGXII minimal medium containing 2% (w/v) glucose. Each graph is showing the average data of three biological replicates including standard deviation. (**B**). Selected mutations, as found in UBm, were step-wise introduced into the wild type strain and their impact on the growth on glucose containing minimal media was examined for three biological replicates. (**C**) The growth of the wild type, UBm and the single mutant strains (WT_corA_Q307 & Δ12 bp) were compared on CGXII minimal medium with 2% (w/v) glucose and different MgSO_4_ concentrations (0.05 mM, 1 mM and 10 mM). Pre-cultivations were performed as described in the material and methods part. Each curve represents three biological replicates including standard deviation.

Remarkably, comparative growth analysis revealed a significant positive effect of all tested *pyk* and *fruK* mutations, whereas single mutations in the *corA* gene showed no significant effect (Q307*) or resulted in a reduced growth rate (12 bp deletion) on standard CGXII minimal medium (Fig. 5A). The combination of *pyk* A271T and *fruK* R71L (as identified in strain UBm) led to an even higher growth rate on glucose demonstrating the synergistic effect of these key targets (Fig. 5B). However, the introduction of the *corA* mutation (*corA*_Δ12 bp, as found in UBm) in the double mutant (*pyk* A271T and *fruK* R71L) had no or only minor effects on the growth (standard CGXII) in comparison to the double mutant (Fig. 5B). This is in line with the finding that the restoration of the native *corA* gene in UBm did not show a significant effect either (Figure S7). Since none of the tested mutants reached the fitness level of the isolated UBm strain (Table 3, Fig. 5A,B), it is likely to assume that epistatic interactions between further key mutations improve adaptation to growth on glucose.

As the *corA* gene encodes a putative Mg^2+^ importer, we also tested the impact of Mg^2+^ excess (10 mM MgSO_4_) and limitation (0.1 mM MgSO_4_). Remarkably, a reduction of Mg^2+^ availability led to a significant growth advantage of strains carrying one of the two *corA* mutations tested. Both mutants (Q307* and Δ12 bp) displayed a faster growth within the exponential phase but appeared to have a lower biomass yield than the wildtype strain (Fig. 5C).

Further analysis of *pyk* mutations *via in vitro* enzyme assays revealed a significantly decreased pyruvate kinase activity for the single mutants T12A and A20V and the strain UBw (containing A20V). In contrast, no difference in pyruvate kinase activity was measured for A271T (identified in UBm) under the tested conditions. Remarkably, this mutant showed the strongest impact on growth (Table 3). Analysis of glucose consumption rates of evolved and parental strains revealed an increase of up to 40% reached by the *C. glutamicum* MB001 derived strain UBm (Table 3). In addition, the *pyk* A271T and *fruK* R71L mutants displayed increased consumption rates, whereas *pyk* T12A mutation did not significantly impact glucose import (Table 3). This effect on glucose uptake was also supported by the analysis of a promoter fusion of *ptsG* to *eyfp*, which revealed an increased *ptsG* expression in UBw and UBm as well as in strains carrying *pyk* or *fruK* mutations (Table 3, Figure S8). The *ptsG* gene encodes the enzyme II of the phosphotransferase system representing the major transport system involved in glucose uptake in *C. glutamicum*.

### Evolutionary trade-offs - Utilization of different carbon sources

**Figure 6:**
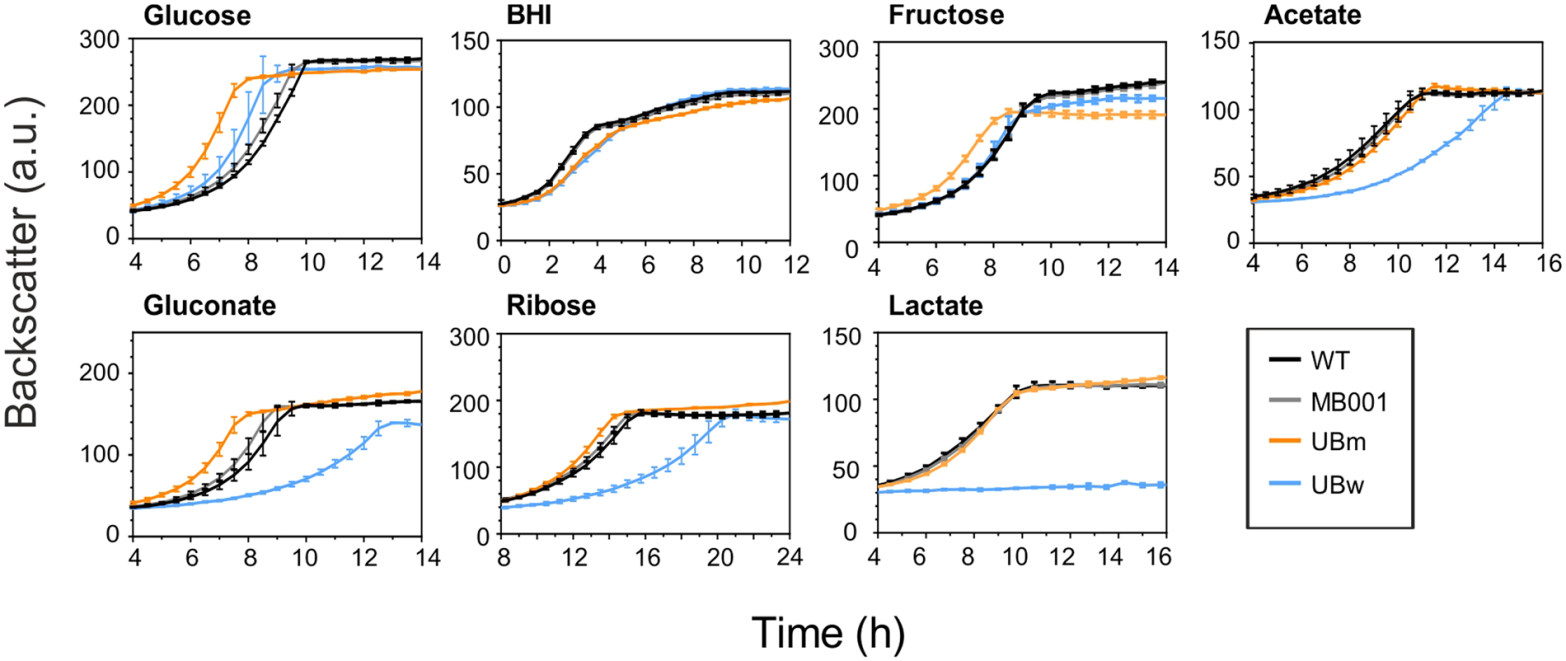
Analysis of trade-off effects of evolved strains growing on different carbon sources. The growth of *C. glutamicum* UBm, UBw and the parental strains was analyzed on CGXII minimal medium supplemented with different carbon sources: glucose 2% (w/v), BHI (37 g/L), fructose 2% (w/v), gluconate 2% (w/v), ribose 2% (w/v), lactate 1.5% (w/v) and acetate 1.5% (w/v). For each condition three biological replicates were analyzed.

In the described ALE experiment, we selected for strains harboring mutations that improve the fitness of *C. glutamicum* on glucose minimal medium. However, the missing selective pressure may tolerate the fixation of mutations detrimental for the catabolism of other carbon sources. Here, we investigated the growth of UBw and UBm on CGXII minimal medium containing fructose, gluconate, ribose, lactate or acetate as sole carbon sources. In addition, we also tested growth on BHI complex medium (Fig. 6). Evolved strains already displayed a slightly decelerated growth rate on BHI medium reflecting the systemic adaption to minimal media. On gluconate, ribose and acetate, UBw showed a strongly impaired growth in comparison to the parental strains. Growth on lactate was even totally abolished due to a transposon insertion into the lactate permease gene (Table S6). Referring to previous studies, a polar effect of the transposon on downstream lactate dehydrogenase gene may be responsible for the loss of lactate utilization^35^. In contrast, UBm cells revealed a competitive or even improved growth on all tested carbon sources and under osmotic stress conditions (Figs 6 and S3C).

As a next step, we investigated the effects of key mutations on the utilization of different carbon sources, including fructose, gluconate and in case of *fruK* also sucrose. Whereas the *fruK* T6I mutation led to a strong growth defect on fructose, R71L slightly improved the fitness. However, the T6I mutation caused a marginal lower biomass formation on sucrose where R71L mutants showed a comparable behavior as the wild type strain (Figure S9). In the *pyk* mutants, A20V and T12A, growth on gluconate was strongly impeded, but the A271T mutant was not affected. In contrast, all *fruK* mutations caused a significant improvement when gluconate served as sole carbon source (Table 3, Figure S9). These results provide only a brief glimpse on the systemic changes of the evolved strains clearly affecting a variety of different metabolic routes and cellular processes.

## Discussion

The basic principles of evolution, mutation and selection, are the unbeaten drivers of microbial adaptation^13^. In this study, we compared the potential of the biotechnological platform organism *C. glutamicum* ATCC 13032 and its prophage-free variant MB001 to evolve towards higher growth rates on glucose minimal medium. Sequencing of two independent cell lines for both strains (ATCC 13032) as well as the genomes of isolated top strains revealed key mutations causing accelerated growth under the chosen conditions. Both strains showed a comparable adaptive behavior and already in the first 100 generations an improvement in fitness by about 20%. In Lenski’s famous long-term experiment an increase of about 20% was achieved after ~1000 generations^36^. Thus, our experiment reveals a high potential of *C. glutamicum* to evolve towards higher growth rates on defined media. In another study, where *E. coli* cells were kept constantly in the exponential phase, growth rates improved even faster and after ~2000 generations an overall increase over 50% was observed^2^. Key mutations in *pyk, fruK* and *corA*, which occurred independently in *C. glutamicum* in different cell lines and both strains, were identified as causative mutations for higher growth rates on glucose. The overall approach resulted in the isolation of two top strains UBw (ATCC 13032 derivative) and UBm (from MB001), which were characterized in further detail. Here, the positive features of the strain UBm deserve special emphasis. This strain, derived from the prophage-free variant MB001, showed an increase in fitness of about ~28% and displayed – in contrast to UBw - almost no significant trade-off or even better growth on the other carbon sources tested (Fig. 6 and S3).

A previous study on *Escherichia coli* K12 revealed a high importance of its cryptic prophage elements on growth and stress resistance^18^. However, we observed no significant differences in the adaptive behavior, mutation frequency or transposon activity of *C. glutamicum* ATCC 13032 wild type and its prophage-free derivative MB001 in our comparative ALE experiment (Figure S6, Table S7). Furthermore, all cryptic prophage elements were stably maintained throughout the ALE of the wild type strain. Although a small fraction of cells is continuously killed by the spontaneous activation of the cryptic prophage (CGP3)^28,29^ this burden has apparently no strong impact on the fitness under the chosen conditions. A reason for this is likely the presence of the nucleoid-associated protein CgpS encoded in the prophage CGP3 and acting as a silencer of phage gene expression^37^. CgpS, therefore, inherits a central role in the maintenance of foreign DNA elements in the host genome.

The *pyk* gene, encoding pyruvate kinase (PK), was found to represent a key genetic region for an improvement of growth on glucose in *C. glutamicum*. This is in line with evolutionary studies of *E. coli*, where also *pyk* mutations were identified^2,38^. Barrick *et al*. showed that re-introduction of *pyk* mutations into the wild type background improved growth rates and suggested that these mutations cause a decrease in PK activity^1,39^. Lower enzymatic activity of PK would consequently lead to a higher PEP pool driving the phosphotransferase system (PTS)^38,39^. Also in eukaryotes, a catalytically less active PK isoform is found in highly proliferating cells like embryonic stem or cancer cells^40^. In fact, it is suggested that the low-active isoform PKM2 plays a key role in the metabolic reprogramming causative for the so-called Warburg effect observed in cancer cells, where large amounts of glucose are exploited for anabolic reactions^41^. These data are in agreement with our study, as we confirm the positive effect of *pyk* mutations by reintroducing selected SNPs into the wild type background. Furthermore, we showed that two of the mutations (T12A, A20V), in fact, led to a decrease in PK activity, whereas the third mutation A271T did not significantly alter the PK activity in the *in vitro* assay (Table 3). Contrary to the suggested hypothesis, we have not observed an increased glucose uptake rate for strains harboring a less active PK, but for the A271T mutant (Table 3). Notably, a simple in-frame deletion of *pyk* did not result in significant differences in comparison to the wild type regarding growth or glucose consumption as reported by previous studies^42,43^. Comparing PK amino acid sequence of *C. glutamicum* to the published structure of *E. coli* ^44^ (sequence identity of 41%) revealed that the two residues T12 and A20 are located in close vicinity to the catalytic center. This finding is in agreement with the observed decrease in enzymatic activity. Strikingly, residue A271 was a subject of another study where mutagenesis of this respective amino acid resulted in decreased PK activity upon increased levels of the allosteric activator fructose 1,6-bisphosphate (FBP)^45^. Hence, we conclude that PK is indeed an important target to enhance cellular proliferation – from bacteria to eukaryotes.

**Figure 7:**
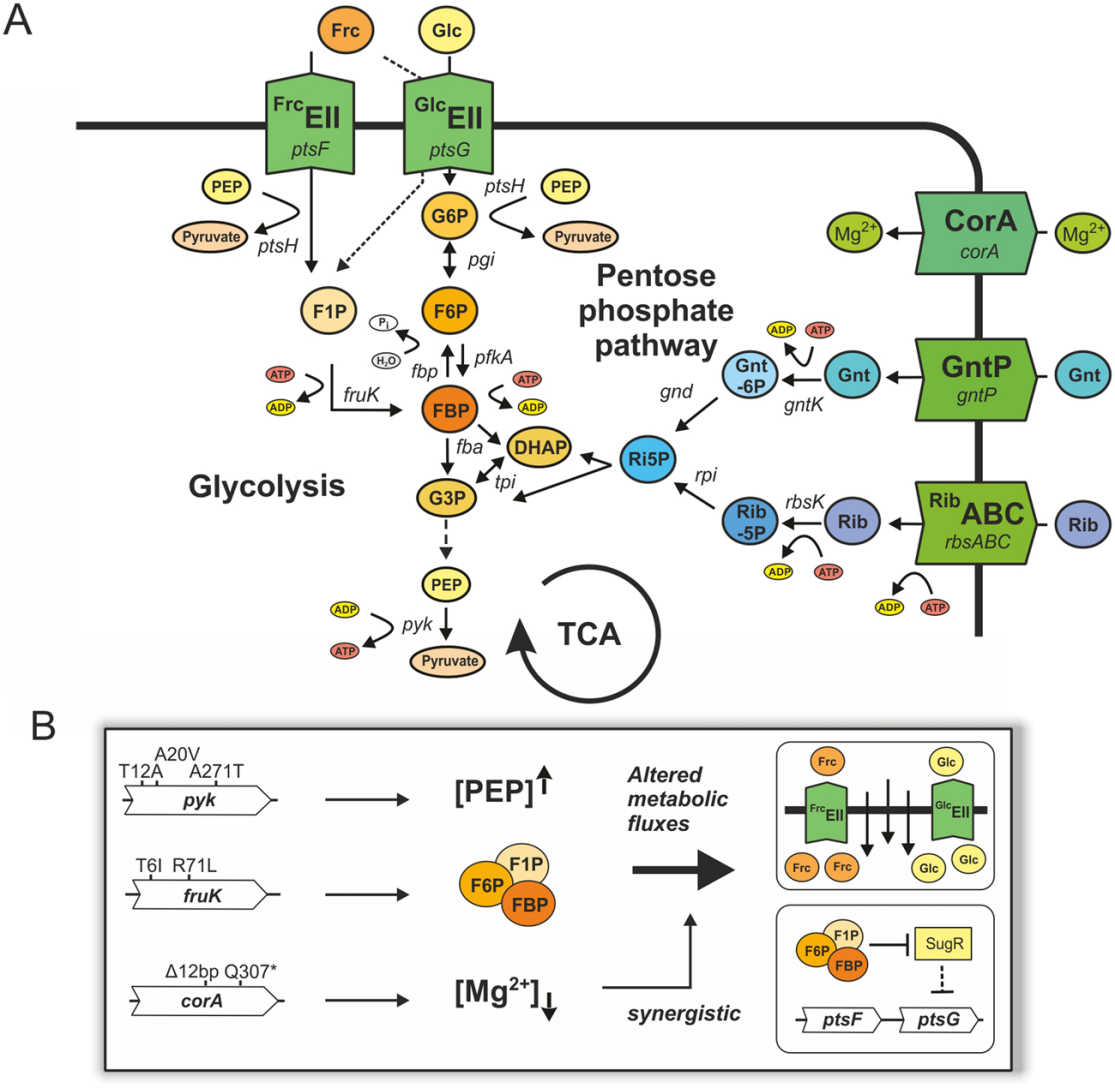
Impact of key mutations on central carbon metabolism in *C. glutamicum*. (**A**) Overview of the metabolic context of identified key mutations, including glycolysis, pentose phosphate pathway (PPP) and tricarboxylic acid cycle (TCA). Abbreviations: Glc: glucose, Frc: fructose, Gnt: gluconate, Rib: ribose, G6P: glucose 6-phosphate, F6P: fructose 6-phospahte, FBP: fructose 1,6-bisphosphate, G3P: glyceraldehyde 3-phosphate, DHAP: dihydroxyacetone phosphate, PEP, phosphoenolpyruvate, Gnt-6P: gluconate 6-phosphate, Rib5P: ribose 5-phosphate, Ri5P: ribulose 5-phopshate, ^Glc^EII: glucose-specific enzyme IIB component of the phosphotransferase system, CorA: Mg^2+^ transporter, GntP: gluconate permease, ABC: Rib-ATP: ATP dependent ABC-transporter. (**B**) Postulated impact of key mutations in *pyk*, *fruK* and *corA*. We hypothesize that mutations in *pyk* and *fruK* both contribute to an elevated glucose uptake by increasing the PEP pool (*pyk* mutations) or by an accumulation of hexose phosphates (*fruK*) relieving SugR repression. Finally, our data suggest that mutations in *corA* (Mg^2+^ import) are likely epistatic and may act synergistically to increase glycolytic flux by reducing intracellular Mg^2+^ levels.

A further player of the central metabolism, which was affected in several independent cell lines, was the *fruK* gene, encoding the 1-phosphofructokinase (Pfk1). Pfk1 catalyzes the transfer of a phosphoryl group from ATP to fructose 1-phosphate (F1P) yielding FBP (Fig. 7A). This reaction is supposed to be mainly relevant for fructose catabolism. However, since fructose was absent in this experiment a certain promiscuity of Pfk1 or at least a high potential to evolve other catalytic functions can be speculated. It is noteworthy that although *fruK* mutations were not identified in the *E. coli* ALE study, this gene was highly upregulated in their evolved strains^2^. Introduction of the two *fruK* mutations, T6I and R71L, into our parental wild type strain resulted in a significant fitness increase (Table 3, Fig. 5A). Growth experiments on fructose minimal medium revealed a negative impact of the T6I mutation (Figure S7) comparable to a *fruK* deletion strain^46,47^. In contrast, R71L mutants acquired benefits by surpassing slightly the growth of the parental strain (Table 3, Figure S9). Considering the crystal structures available for *E. coli* Pfk^48^ suggests that both SNPs are not close to the active site but may result in conformational changes. In recent studies, a link between *fruK* deletion and an increase in glucose uptake was indeed described for *C. glutamicum* ^46,47^. The authors suggested that *fruK* deletion results in an increased pool of F1P or in other hexose phosphates relieving SugR repression of *ptsG* ^46^. SugR is a pleiotropic transcriptional repressor of PTS genes and sugar phosphates including F1P, FBP and glucose-6-phosphate (G6P)^49^ were described as potential effector molecules leading to the de-repression of PTS targets. In line with this, an increased expression of *ptsG* and a higher glucose uptake rate was observed for the *fruK* R71L mutant (Table 3). As fructose was not added to the growth medium, one can assume that this effect is unlikely a result of F1P accumulation, but may be caused by increased levels of other hexose phosphates (Fig. 7). However, as shown by Wang *et al*., in a *fruK ptsF* double knockout strain F1P is still detectable indicating the formation of F1P by unknown side reactions e.g. due to activities of phosphohexomutases^46^.

Remarkably, the highest number of different mutations was identified in the *corA* gene, encoding a putative CorA-like Mg^2+^/Co^2+^ transporter protein (Table 1). CorA transporters are beside MgtE the primary transporters of Mg^2+^ in bacteria^50^. Both classes of transporters are identified in the genome of *C. glutamicum* (CorA: cg0080, MgtE1 and MgtE2: cg0275 and cg1276) and appear to be similar in their mode of action by using an electrochemical gradient for the transport of Mg^2+^ ions^50^. However, in this experiment, mutations were only found in the *corA* gene (Table S6). Two transpositions, two nonsense mutations (W365*, Q307*) and a deletion of 12 bp were identified - strongly suggesting a loss of the CorA function. Under standard conditions, neither the introduction of *corA* mutations in the *pyk*/*fruK* double mutant (*pyk* A271T *fruK* R71L) nor the restoration of the wildtype *corA* in the UBm strain led to a significant growth effect. However, cultivation under Mg^2+^ limiting conditions revealed a significantly increased growth rate of the single *corA* mutations (*corA* Q307* and Δ12 bp) as well as for the strain UBm (Fig. 5C). Hence, this benefit explains the high number of mutations that appeared in this genomic region and their fast fixation rates (Table S4, Fig. 4). Also in the *E. coli* ALE experiment of LaCroix *et al. corA* mutations were identified, but the impact on glucose uptake was not elucidated^2^. Recently, it was shown that extracellular magnesium concentrations are directly linked to glucose consumption^51^. As a further piece of evidence, *E. coli* cells displayed faster glucose consumption and higher metabolic flux in a magnesium limitation experiment^52^. Altogether, these findings suggest that reduced CorA activity may also act by increasing metabolic flux on glucose, thereby conferring a competitive advantage of clones carrying a *corA* mutation in the ALE experiment. Furthermore, it is likely to assume that so far uncharacterized epistatic interactions between the genomic variants significantly contribute to an improved fitness of the evolved populations.

In summary, our study reports on the first comparative evolution experiment using the wild type strain and its prophage-free variant and highlights their strong metabolic adaptability. We describe key mutations in *pyk*, *fruK* and *corA* which cause a significant increase of the growth rate on glucose minimal medium or under Mg^2+^ limiting conditions in the case of *corA*. The experiment resulted in the isolation of the top strains UBw (based on ATCC 13032) and UBm (MB001 derivative), where especially the MB001 derived strain displays several positive features and no obvious trade-off under the conditions tested in this study. UBm represents to date the *C. glutamicum* strain with the fastest growth rate on glucose minimal medium and, thus, provides a strong basis for future metabolic engineering approaches. The fact that similar genetic regions were affected in independent studies with the model strains *E. coli* and *C. glutamicum* also highlights the broad significance of these data for the understanding of microbial metabolic networks and for the identification of major bottlenecks for metabolic flux. The identification and further analysis of causative key mutations is not only relevant for future metabolic engineering but also enhances our understanding of biological systems.

## Material and Methods

### Bacterial strains and growth conditions

Bacterial strains used in this study are listed in Table S1. *C. glutamicum* ATCC 13032 served as wild type strain^53^. Strain MB001 is the prophage-free variant of ATCC 13032^19^. *E. coli* DH5α was used for cloning and was cultivated at 37 °C in lysogeny broth (LB) (if not indicated otherwise). *C. glutamicum* cells were grown in complex medium consisting of Brain-Heart-Infusion (BHI, 37 g/L) (DifcoTM BHI, Becton, Dickinson and Company (BD)) or in minimal medium CGXII^54^ with 30 mg·L^−1^ biotin. Unless stated otherwise, 2% (w/v) glucose was added as carbon source. For growth experiments, *C. glutamicum* cells from a fresh BHI agar plate were cultivated for 6 h in BHI at 30 °C. Subsequently, this preculture was used to inoculate a second preculture in CGXII minimal medium with 2% (w/v) glucose which was incubated at 30 °C overnight. On the next day, the main culture was inoculated with cells of the 2nd preculture in defined CGXII media. If necessary, kanamycin was added in a final concentration of 50 µg·mL^−1^ for *E. coli* and 25 µg·mL^−1^ for *C. glutamicum*.

### Recombinant DNA work

Plasmids and oligonucleotides used in this work are specified in Table S2. Standard cloning methods including PCR, restriction and ligation of DNA were conducted according to established protocols^55^. Cloning of plasmids was conducted using Gibson assembly^56^. Synthesis of oligonucleotides and DNA sequencing were performed by Eurofins MWG Operon. The chromosomal integration of the barcode sequences, the mutations *pyk*_T12A, *pyk*_A20V, *pyk*_A271T, *fruK*_T6I, *fruK*_R71L and *corA*_Q307* and the deletion of 20 bp within the *corA* gene were performed via the two-step homologous recombination method^57^. Approx. 500 bp of the up- and downstream region of the replacement site were amplified by PCR using oligonucleotides listed in Table S3. Integration of the 20 bp barcode sequences were checked by colony-PCR with the oligonucleotides WT_BC_fw and WT_BC_rv in the wild type strain and with MB_BC_fw and MB_BC_rv in the prophage-free MB001 strain. Insertions of the mutations and of the 12 bp deletion in *corA* were verified by sequencing applying the oligonucleotides pyk_T12A-seq_fw, pyk_T12A-seq_rv, pyk_A271T-seq_fw, pyk_A271T-seq_rv, *corA*_del_seq_fw, *corA*_del_seq_rv, fruK_T6I_seq_fw and fruK_T6I_seq_rv.

### Adaptive laboratory evolution experiment

The adaptive evolution experiment was conducted with genomically barcoded ATCC 13032 and prophage-free MB001 strains. The cultivations were performed in 48-well FlowerPlates® at 30 °C and a shaking frequency of 900 rpm in a microtron cultivation system (Infors HT). The first and second precultivation were done as described in the bacterial strains and growth conditions section. The evolution experiment started with six biological replicates of each strain and an initial cell density (OD_600_) of 1. Standard growth conditions were chosen for the cultivations by using the minimal medium CGXII containing 2% (w/v) glucose. To keep differences in medium composition to a minimum, CGXII was prepared in sufficient amounts at the beginning of the experiment, aliquoted and stored at −80 °C. Every 48 h to 72 h 10 µl of stationary cells were used to inoculate 790 µl of fresh medium (dilution 1:40) in the microtiter plate. In total, 90 serial transfers were performed during this ALE experiment. At defined time points (approx. every 15th transfer) samples were taken and stored as glycerol cultures.

For the first twenty cultivations, standard CGXII medium was alternated with iron depleted (1 µM Fe^2+^) CGXII medium in order to investigate potential effects of spontaneous prophage induction stimulated by alternations in iron availability (data not shown). Since, this did not result in any significant differences in growth or stress resistance between the wild type and MB001, this step was avoided in further transfers (data not shown) (final 70 transfers to standard CGXII medium).

Number of generations were calculated throughout the experiment by estimating a start OD of about ~0.5 and a final OD_600_ = 35–40 resulting in approx. 6–7 generations for each cultivation.

To reproduce the observed fitness leap after ~100 generations, a repetition of the early phase of the ALE experiment was performed. Here, 16 serial transfers in standard CGXII medium containing 2% (w/v) glucose were conducted every 24 h in three biological replicates. Samples for glycerol cultures were taken every 2nd cultivation.

For comparative studies (e.g. by genome sequencing and fitness tests), two cell lines that originated from different biological replicates of the two strains were used from the first (100 and 140 generations) and last two time points (540 and 630 generations). Moreover, from the repeated ALE experiment one cell line of each strain were examined after 50 and 80 generations.

The growth characterization of evolved and re-engineered strains were conducted using the BioLector® microcultivation system of m2p-labs as described in^58^. 48-well FlowerPlates® were applied for the cultivation in 750 µl of defined CGXII media with 2% (w/v) glucose (or alternative carbon source) at 30 °C and 1200 rpm shaking frequency. Typically, cultures were inoculated to a start OD_600_ of 1, unless specified otherwise. Measurements were taken every 15 minutes.

### Genome sequencing

Genomic DNA of *C. glutamicum* cells was purified using the NucleoSpin® Microbial DNA Kit (Macherey Nagel). About ~100 mg cell pellet (from ~1 ml BHI-overnight culture) yielded approx. 10 µg DNA. In total, 4 µg of genomic DNA was used for library preparation and indexing with the TruSeq DNA PCR-free sample preparation kit (Illumina). Quantifications of the resulting libraries were conducted using KAPA library quant kits (Peqlab) and were normalized for pooling. A MiSeq sequencing device (Illumina) was used for paired-end sequencing with a read-length of 2 × 150 bases. Data analysis and base calling were accomplished with the Illumina instrument software and stored as fastq output files. Obtained sequencing data were imported into CLC Genomics Workbench (Qiagen Aarhus A/S) for trimming and base quality filtering. The output was mapped to accession BX927147 as the *C. glutamicum* ATCC 13032 reference genome^34^ or CP005959 as the reference genome for MB001^19^. The resulting mappings were used for the quality-based SNP/variant detection with CLC Genomics Workbench. The detected SNPs were manually inspected regarding their relevance.

### Dilution drop stress tests

*C. glutamicum* cells of an BHI overnight culture were diluted to an OD_600_ = 1 in phosphate buffered saline (PBS) solution (137 mM NaCl, 2.7 mM KCl, 20 mM Na_2_HPO_4_, 1.8 mM KH_2_PO_4_). Eight serial dilutions (10^0^–10^−7^) were prepared and 3 µl were spotted on CGXII agar plates containing 2% (w/v) glucose. Before spotting on agar plates, cells were exposed to different stresses (heat stress: 1 h at 42 °C; UV stress: UV light exposure for 1 or 5 min (254 nm, 6 W); osmotic stress: agar plate containing 1 M NaCl). If not indicated otherwise, plates were incubated for 48 h at 30 °C.

### Flow cytometry

Flow cytometry was used to determine the ratio between eYFP and E2-Crimson positive cells during the competitive growth experiment of ATCC 13032 and MB001 (Fig. 1). Measurements and sorting were performed with a FACS Aria II (BD). A blue solid state laser with an excitation wavelength of 488 nm (to excite eYFP) and a red gas laser for excitation at a wavelength of 633 nm (to excite E2-Crimson) were used. Cytometer set-up, measurements and sorting were conducted as described previously^29^.

### Determination of glucose uptake rate

Glucose concentrations of culture supernatants were determined by using the D-Glucose Kit (Roche) according to manufactory’s protocol. At defined time points, 500 µl samples were taken (Figure S3) and cells were harvested by centrifugation (16,000 *g*, 3 min). The obtained supernatant was directly used for the assay or stored at −20 °C. Uptake rates were calculated as described in Frunzke *et al*.^59^ by the following equation:

1 $$\frac{S}{M}\times \mu [\frac{mmol\,\cdot {L}^{-1}\,\cdot O{D}^{-1}}{gDW\,\cdot {L}^{-1}\,\cdot O{D}^{-1}}\cdot {h}^{-1}]=[\frac{mmol}{gDW\,\cdot h}]$$

S is the slope of a regression line which was calculated by plotting the glucose concentrations against the optical density at 600 nm OD_600_. M describes the correlation between dry weight and OD by assuming an OD_600_ of 1 corresponds to 0.25 g dry weight per liter. The growth rate µ was calculated as described in following section.

### Determination of growth rate

Based on the growth curves obtained by cultivations in the BioLector® system or by shaking flask experiments growth rates were calculated using R^60^ by applying following exponential fit to the exponential growth phase.

2 $$N(t)={N}_{0}\ast \,{e}^{-\mu \cdot t}$$

In this equation µ describes the growth rate, t is the time, N(t) population size at time point t and N_0_ represents initial population size. Prior to the exponential fit, measured backscatter data were corrected by subtracting background values, which were defined as backscatter values measured after the first time point (t = 15 min).

### Pyruvate kinase assay

The activity of the pyruvate kinase was measured in cell crude extracts using the Pyruvate Kinase Activity Assay Kit (Sigma). This enzymatic assay is based on the coupling with a pyruvate oxidase. The product is proportional to formed pyruvate and can be measured at 570 nm. Measurements were conducted using an Infinite 200 PRO reader (Tecan). Crude extracts were prepared of cells that were harvested in mid-exponential phase at an OD_600_ ~ 5.

### Data availability statement

All data generated or analysed during this study are included in this published article (and its Supplementary Information files) or will be provided by the corresponding authors upon request.

### Importance

*Corynebacterium glutamicum* represents one of the most important industrial platform strains used for the large-scale production of amino acids (>5 million tons of glutamate and lysine per year) and proteins. Intensive metabolic engineering resulted in a multitude of strains producing further value-added products such as organic acids, polymer precursors and secondary metabolites. Glucose is still the most prominent substrate used for biotechnological production processes. In this study, we evolved *C. glutamicum* ATCC 13032 and its prophage-free variant MB001 towards accelerated growth rates on glucose minimal medium. Genome sequencing of isolated top strains and the analysis of the evolutionary trajectories of evolved populations resulted in the identification of key mutations improving growth on glucose. These data do not only provide an important basis for future metabolic engineering approaches, but also enhance our understanding of biological systems.

## Electronic supplementary material

Supplementary information

Table S6

Table S7
