# Supplementary material for: Adaptive laboratory evolution of Corynebacterium glutamicum towards higher growth rates on glucose minimal medium
Source: Sci Rep. 2017 Dec 1;7:16780. doi: 10.1038/s41598-017-17014-9 (PMC5711897; doi:10.1038/s41598-017-17014-9)
Supplement: Supplementary file 1 — Supplementary information [file 41598_2017_17014_MOESM1_ESM.pdf]

Supplementary material to

Adaptive laboratory evolution of *Corynebacterium glutamicum* towards higher growth rates on glucose minimal medium

Eugen Pfeifer, Cornelia Gätgens, Tino Polen*, and Julia Frunzke*

Institute of Bio- und Geosciences, IBG-1: Biotechnology, Forschungszentrum Jülich GmbH,

52425 Jülich, Germany

*Corresponding author:

Julia Frunzke; Email: j.frunzke@fz-juelich.de; Phone: +49 2461 61 5430

Tino Polen; Email: t.polen@fz.-juelich.de; Phone +49 2461 61 6205

Supplementary Tables

Table S1: Bacterial strains used in this study.

Strains	Relevant characteristics	Reference
E. coli		
DH5α	*supE44* Δ*lacU169* (φ80*lacZ*DM15) *hsdR17 recA1 endA1 gyrA96 thi-1 relA1.*	Invitrogen
C. glutamicum		
ATCC 13032	Biotin-auxotrophic wild type.	1
MB001	ATCC 13032 with in-frame deletion of prophage CGP1, CGP2 and CGP3	2
Wildtype_bc	Wild type derivative; To barcode the strains the sequence 5'GCGCTTCATATGGTCAGTTATGAAG-3' was integrated into the locus between cg1121 and cg1122	This study
MB001_bc	MB001 derivative; the barcode 5'CATTCC AAGCTT GATCCGTACATAG-3' was integrated into the intergenic region between cg1121 and cg1122	This study
ATCC 13032::Ptac-eyfp	Wild type derivative; contains an *eyfp* gene which is under the control of *tac* promoter and which were integrated into the region between cg1121-cg1122	2
ATCC 13032::Ptac-crimson	Wild type derivative; contains an *crimson* gene which is under the control of *tac* promoter and which were integrated into the region between cg1121-cg1122	2
MB001::Ptac-eyfp	MB001 derivative; contains an *eyfp* gene which is under the control of *tac* promoter and which were integrated into the region between cg1121-cg1122	2
MB001::Ptac-crimson	MB001 derivative; contains an *crimson* gene which is under the control of *tac* promoter and which were integrated into the region between cg1121-cg1122	2
ATCC 13032 pyk_T12A	Wild type derivative; in the *pyk* gene (cg2291) adenine at the position 34 was changed to guanine	This study
ATCC 13032 pyk_A20V	Wild type derivative; in the *pyk* gene (cg2291) cytosine at the position 34 was changed to adenine	This study
ATCC 13032 pyk_A271T	Wild type derivative; in the *pyk* gene (cg2291) guanine at the position 811 was changed to adenine	This study
ATCC 13032 fruK_T6I	Wild type derivative; in the *fruK* gene (cg2119) cytosine at the position 17 was changed to adenine	This study
ATCC 13032 fruK_R71L	Wild type derivative; in the *fruK* gene (cg2119) guanine at the position 212 was changed to adenine	This study
ATCC 13032 corA_del	Wild type derivative; in gene *corA* the 12 bp CGTCGACGATGG position 593 to 604 in cg0080	This study
ATCC 13032 corA _Q307*	Wild type derivative; in gene *corA* cytosine at the position 919 was changed to thymine	This study
ATCC 13032 pyk_A271T_fruK_R 71L	Wild type derivative; in *pyk* gene (cg2291) guanine at the position 811 was changed to adenine and in *fruK* guanine at the position 212 was changed to adenine	This study
UBm_*corA*	UBm derivative; mutated *corA* gene (cg0080), in which 12bp were deleted during the ALE experiment, was replaced by the wild type *corA* gene	This study

Table S2: Plasmids used in this study.

Plasmid	Relevant characteristics	Reference
pK19mobsacB	plasmid that is used for allelic exchange in *C. glutamicum*; *oriV*$_{E.c.}$, *sacB lacZα KanR, Eco*RI and *Hin*dIII restrictions sites were for Gibson assembly	[3]
pK18mobsacB-int1	KanR; plasmid is used for integration of DNA into the intergenic region between cg1121 and cg1122 (*oriV*$_{E.c}$ *sacB lacZ*)	[2]
pK18_BC_WT	derivative of pK18 that was used to barcode (5'GCGCTTCATATGGTCAGTTATGAAG-3') the wild type strain	This study
pK18_BC_MB	derivative of pK18 that was used to barcode (5'CATTCCAAGCTTGATCCGTACATAG-3') the MB001 strain	This study
pK19_pyk_T12A	derivative of pK19 to replace native *pyk* gene with *pyk*_T12A	This study
pK19_pyk_A20V	derivative of pK19 to replace native *pyk* gene with *pyk*_A20V	This study
pK19_pyk_A271T	derivative of pK19 to replace native *pyk* gene with *pyk*_A271T	This study
pK19_fruK_T6I	derivative of pK19 that was used to insert the mutation T6I into the *fruK* gene	This study
pK19_fruK_R71L	derivative of pK19 that was used to insert the mutation R271L into the *fruK* gene	This study
pK19_ corA _del	derivative of pK19 that was used to delete 18 bp of the *corA* gene	This study
pK19_ corA _Q307*	derivative of pK19 that was used to insert the mutation Q307* in *corA*	This study
pK19_corA	derivative of pK19 that was used to replace the mutated *corA* gene in the UBm strain	This study
pJC1	Kan^R, Amp^R, *C. glutamicum* and *E. coli* shuttle vector	[4]
pJC1-*ptsG-eyfp*	pJC1 derivative containing promoter of *ptsG* fused to *eyfp*. 657 bp upstream of cg1537 (*ptsG*) were considered as promoter region.	This study

Table S3. Oligonucleotides used in this study. Bold sequences point out overlapping parts for Gibson Assembly.

Oligonucleotide	Sequence (5' -> 3')	Comment
WT_OL_fw	**TCCTCTTGCTCGTGTCAATT**GCGCTTCATATGGTCAGTTATGAAG	Label of wild type strain
WT_OL_rv	**GTGTCCATGAGTTCGCTCGA**CTTCATAACTGACCATATGAAGCGC	
WT_BC_fw	GCGCTTCATATGGTCAGTTATGAAG	
WT_BC_rv	CTTCATAACTGACCATATGAAGCGC	
MB001_OL_fw	**TCCTCTTGCTCGTGTCAATT**CATTCCAAGCTTGATCCGTACATAG	Label of MB001 strain
MB001_OL_rv	**GTGTCCATGAGTTCGCTCGA**CTATGTACGGATCAAGCTTGGAATG	
MB001_BC_fw	CATTCCAAGCTTGATCCGTACATAG	
MB001_BC_rv	CTATGTACGGATCAAGCTTGGAATG	
		Construction of
pyk_T12A-P1	**GACCATGATTACGCCAAGCT**ACCTTGGGCCGTTTCTGG	pK19_pyk_T12A
pyk_T12A-P2	**GCCACCGCTGGGCCTAGGGC**ACATACAATCTTAGTTCGTCTATCCAC	
pyk_T12A-P3	**GAACTAAGATTGTATGTGCC**CTAGGCCCAGCGGTG	
pyk_T12A-P4	**AAAACGACGGCCAGTGAATT**AGGTCACGGATATCCTTTTCGG	
pyk_T12A-seq_fw	ACCTTGGGCCGTTTCTGG	
pyk_T12A-seq_rv	AGGTCACGGATATCCTTTTCGG	
pyk_A20V-P2	**AAACGCAGAATTCCATCTAC**ACTAGCCACCGCTGGG	pK19_pyk_A20V
pyk_A20V-P3	**GCCCAGCGGTGGCTAGTGTA**GATGGAATTCTGCGTTTGGTAGAAG	
pyk_A271T-P1	**GACCATGATTACGCCAAGCT**CACGATCGTGTGTCCACC	pK19_pyk_A271T
pyk_A271T-P2	**GCCACGATAACTGGCTTTGT**GTTCTCACGGGCAATCTGG	
pyk_A271T-P3	**AGATTGCCCGTGAGAACACA**AAGCCAGTTATCGTGGCAACC	
pyk_A271T-P4	**AAAACGACGGCCAGTGAATT**GGCATTGCTAAAAGAGCACGG	
pyk_A271T-seq_fw	ACGATCGTGTGTCCACC	
pyk_A271T-seq_rv	GGCATTGCTAAAAGAGCACGG	
corA_del_P1	**GACCATGATTACGCCAAGCT**CTGTACCTGAAGAGGTTTCTCC	pK19_ corA _del
corA_del_P2	**CTAAGAACGGGGGAGAAGC**CGTCGACAAGCCTATAGGC	
corA_del_P3	**GCCTATAGGCTTGTCGACG**GCTTCTCCCCCGTTCTTAG	
corA_del_P4	**AAAACGACGGCCAGTGAATT**ACTGCGTTTGAAGATCCAGTAC	
corA_del_seq_fw	CGTCGGTACCTTCCTGATTCGGC	
corA_del_seq_rv	CCTGGAATTTCCGAGGCC	
corA_Q307*_P1	**GACCATGATTACGCCAAGCT**CATGAAGCCTCAGGCCATAG	pK19_ corA _Q307*
corA_Q307*_P2	**ATGTCTTCGTTTTGGCGTTA**GGCTACAAGGGTGGCG	
corA_Q307*_P3	**ACGCCACCCTTGTAGCCTAA**CGCCAAAACGAAGACATGAAG	
corA_Q307*_P4	**AAAACGACGGCCAGTGAATT**CAGCTCAAGGAACGTTTCCTC	
pk19_ corA_fw	**GACCATGATTACGCCAAGCT**ATGCCAAAGAATTACGACATCAACGG	pK19_ *corA*
pk19_ corA_rv	**AAAACGACGGCCAGTGAATT**TCACATCCACTTACTGCGTTTGAAGAT	
fruK_T6I_P1	**GACCATGATTACGCCAAGCT**CCTGGATTCAGGTTTAGCGTG	

fruK_T6I_P2	**TCAATACTCGGGTTTGGGAT**GAATGTGATGATCATGGGGTTACC	
fruK_T6I_P3	**CCATGATCATCACATTCATC**CCAAACCCGAGTATTGATTCC	
fruK_T6I_P4	**AAAACGACGGCCAGTGAATT**CCATCAGTGGCTTGTCTGAG	pK19_*fruK*_T6I
fruK_T6I_seq_fw	CTCATCACACCTCTAGCACG	
fruK_T6I_seq_rv	AGCTGGCCCAGTTCCAG	
fruK_R71L_P2	**CGGGCAAGCCGATGTCGAG**GACCAGTGGGACGAAGG	pK19_*fruK*_R71L
fruK_R71L_P3	**CCTTCGTCCCACTGGTC CTC**GACATCGGCTTGCCCG	

Table S4: Frequencies (in %) of mutations in *fruK*, *pyk*, *corA*, *soxA* and *hmuV*, which were identified in the evolved strains. WT_A and WT_B: wild type clones A and B; MB_A, MB_B: MB001 clones A and B.

Gene	Mutation	Strain	#100	#140	#540	#630	Top1	Top2	Top3
fruK	exchange T6I	WT_A	-	-	-	-	-	-	-
		MB_A	-	-	-	20	-	-	-
		WT_B	-	-	-	-	-	-	-
		MB_B	-	-	96	97.73	-	-	-
	exchange R71L	WT_A	-	-	-	-	-	-	-
		MB_A	-	-	-	-	100	-	-
		WT_B	-	-	-	-	-	-	-
		MB_B	-	-	-	-	-	-	-
	Insertion of *tnp13b*	WT_A	-	-	-	-	-	-	-
		MB_A	-	-	-	-	-	-	-
		WT_B	-	-	73	79	-	-	100
		MB_B	-	-	-	-	-	-	-
pyk	exchange A271T	WT_A	-	-	-	-	-	-	-
		MB_A	-	-	91.84	96.43	100	100	99.53
		WT_B	-	-	-	-	-	-	-
		MB_B	-	-	-	-	-	-	-
	exchange P257L	WT_A	-	-	-	-	-	-	-
		MB_A	-	-	-	-	-	-	-
		WT_B	-	-	33.08	37.72	-	-	-
		MB_B	-	-	-	-	-	-	-
	exchange D175G	WT_A	-	14	-	-	-	-	-
		MB_A	-	12.5	-	-	-	-	-
		WT_B	-	-	-	-	-	-	-
		MB_B	-	11.05	-	-	-	-	-
	exchange A20V	WT_A	-	15.38	47.69	28.26	100	100	-
		MB_A	10.67	27.03	-	-	-	-	-
		WT_B	-	-	-	-	-	-	-
		MB_B	12	17.74	-	-	-	-	-
	exchange T12A	WT_A	-	-	53.42	73.81	-	-	-
		MB_A	-	11.43	-	-	-	-	-
		WT_B	-	-	-	-	-	-	-
		MB_B	-	-	-	-	-	-	-

corA	Stop W365*	WT_A	-	-	-	-	-	-	-
		MB_A	-	-	-	-	-	-	-
		WT_B	-	-	-	-	-	-	-
		MB_B	-	-	96.4	97.8	-	-	-
	Stop Q307*	WT_A	-	-	54.47	25	-	99.49	-
		MB_A	-	-	-	-	-	-	-
		WT_B	-	-	-	-	-	-	-
		MB_B	-	-	-	-	-	-	-
	exchange A302P	WT_A	-	-	-	-	-	-	-
		MB_A	-	-	29.13	25.81	-	99.29	-
		WT_B	-	-	-	-	-	-	-
		MB_B	-	-	-	-	-	-	-
	exchange R291H	WT_A	-	-	-	-	-	-	-
		MB_A	-	-	18.63	11.54	-	-	-
		WT_B	-	-	-	-	-	-	-
		MB_B	-	-	-	-	-	-	-
	Deletion 12 bp, CGTCGACGATGG	WT_A	-	-	-	-	-	-	-
	62905..62916	MB_A	-	-	13.58	30.77	98.55	-	-
		WT_B	-	-	-	-	-	-	-
		MB_B	-	-	-	-	-	-	-
	Insertion of *tnp13b*	WT_A	-	-	36	48	72	-	-
		MB_A	-	-	-	-	-	-	-
		WT_B	-	-	76	72	-	-	89
		MB_B	-	-	-	-	-	-	-
	Insertion of *tnp1b*	WT_A	-	-	-	-	-	-	-
		MB_A	-	-	22	-	-	-	73
		WT_B	-	-	-	-	-	-	-
		MB_B	-	-	-	-	-	-	-
soxA	L26L	WT_A	-	-	23.26	25.51	40.1	-	36.03
		MB_A	-	-	-	-	-	-	-
		WT_B	-	-	27.88	24.22	-	-	-
		MB_B	-	-	-	-	-	-	-
	exchange H29N	WT_A	-	-	-	-	-	-	33.33
		MB_A	-	-	-	-	-	-	-
		WT_B	-	-	25.95	26.71	-	-	-
		MB_B	-	-	-	-	-	-	-
	Deletion,C	WT_A	-	-	-	-	-	-	33.56
	1673063	MB_A	-	-	-	-	-	-	-
		WT_B	-	-	25.95	27.44	-	-	-
		MB_B	-	-	-	-	-	-	-
	exchange L271F	WT_A	-	-	-	-	-	-	-
		MB_A	-	-	-	-	-	-	-
		WT_B	-	-	-	-	-	-	-
		MB_B	-	-	27.57	21.55	-	-	-

hmuV	Insertion, G	WT_A	-	-	-	-	-	-	-
	414917^414918	MB_A	-	13.64	-	-	-	-	-
		WT_B	-	-	-	-	-	-	-
		MB_B	-	-	-	-	-	-	-
	Insertion, A	WT_A	11.54	-	-	-	-	-	-
	414923^414924	MB_A	-	15	12.5	-	-	-	-
		WT_B	-	-	-	-	-	-	-
		MB_B	-	-	-	-	-	-	-
	exchange S73Y	WT_A	16.67	15.15	-	-	10.87	-	-
		MB_A	-	19.23	15.15	11.76	-	-	-
		WT_B	-	-	-	-	-	-	-
		MB_B	-	-	-	11.54	-	-	10.77
	exchange D74E	WT_A	14.29	14.29	-	-	-	-	11.11
		MB_A	-	15.15	10.81	-	-	-	-
		WT_B	-	-	-	-	-	-	-
		MB_B	-	-	-	11.11	-	-	-
	exchange A70E	WT_A	13.04	-	-	-	-	-	-
		MB_A	-	14.29	11.43	-	-	-	-
		WT_B	-	-	-	-	-	-	-
		MB_B	-	-	-	-	-	-	-

Table S5: Comparison of mutations found in samples of the 2nd experiment taken after eight (#50 generations) and twelve (#80 generations) cultivation cycles.

	**WT**		**MB001**	
**SNV and Annotation**	**#50 Freq. (%)**	**#80 Freq. (%)**	**#50 Freq. (%)**	**#80 Freq. (%)**
exchange A70E in cg0469, *hmuV*, hemin transport system, ATP-binding protein				10.71
Insertion of transposon *tnp13a* in cg2119, *fruK*		43		
exchange D334Y in cg3213, putative secreted protein			10.87	10.87
exchange M92I in cg2458,*pgp2*, predicted phosphatase, HAD-family (EC:3.1.3.18)				12.5
exchange S73Y in cg0469, *hmuV*, hemin transport system, ATP-binding protein				13.64
exchange V123L in cg2069, *psp1*, putative secreted protein CGP3 region	100	100		
414917^414918_Insertion_G, *hmuV*, hemin transport system, ATP-binding protein				11.54
414923^414924_Insertion_A, *hmuV*, hemin transport system, ATP-binding protein				12.5
MNV, 3078739..3078740_TA_AT, in cg3213(+), putative secreted protein			10.64	10.64
IGR between cg1121 (putative permease of the major facilitator superfamily) and cg1122 (putative secreted protein)	47.95	43.29	47.19	49.48
S270S in cg3197(+), *psp5*, putative secreted protein	100	100		
exchange F26L in cg2807, *tnp11a*, transposase, putative pseudogene		19.75		
exchange Q12H in cg3342, putative secreted protein			10.53	10.53

Supplementary figures

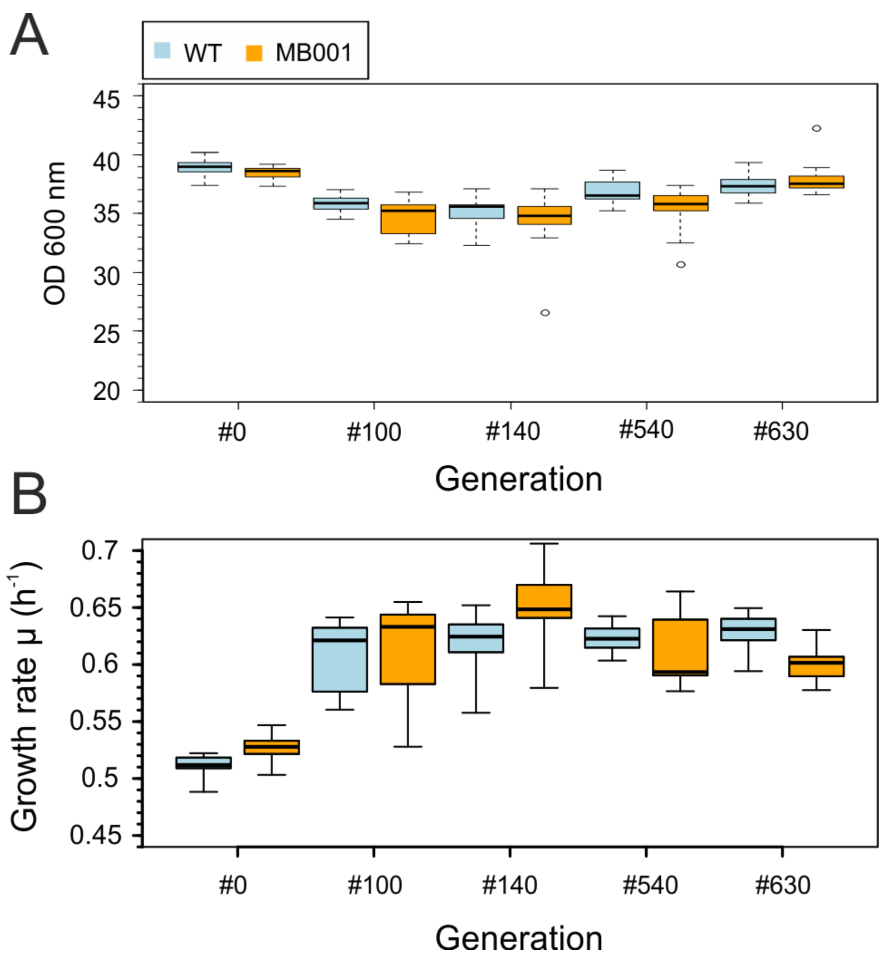

Figure S1: Distributions of growth rates and the final OD_{600} values of cells from the first ALE experiment. Growth studies were conducted as described in material and methods part. **(A)** Final OD values were measured from cells of the first cell line (data of the growth curves are shown in Fig. 3). Outliners are highlighted by a 1.5 IQR presentation of the whiskers of the respective boxplots. **(B)** Growth rates of 24 clones of the two strains (WT and MB001) from different time points of a 2nd initial cell line are compared (as it is shown for the first cell line in Fig.3). Whiskers represent the maximum and minimum values.

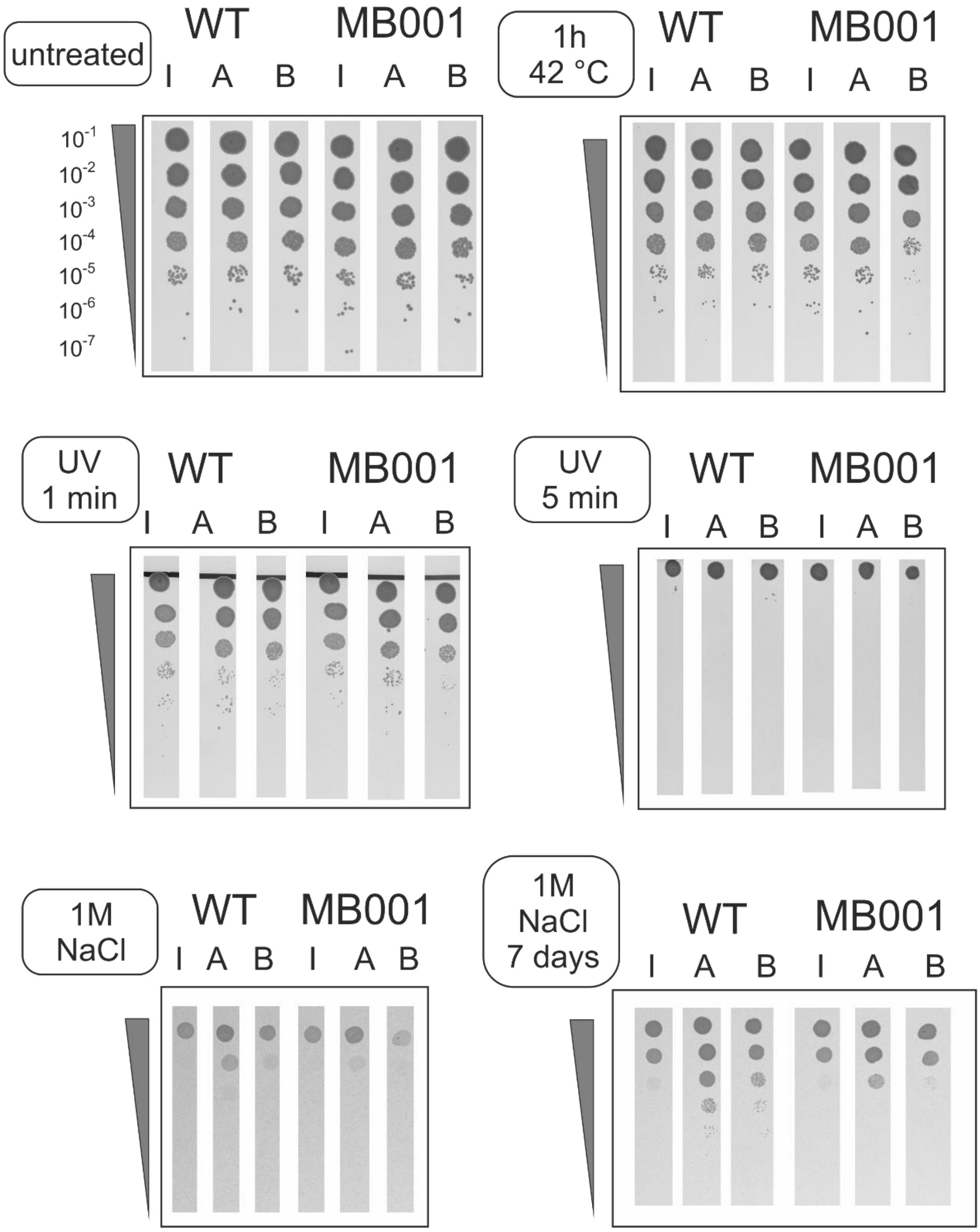

Figure S2: Stress tests of the evolved strains in comparison to the wild type and MB001 strain.
Sensitivity towards stress conditions e.g. heat shock, UV stress and osmotic stress of evolved populations were investigated (630 generations, A = mixture of clone A, B = mixture of B) and initial strains (I). The pre-cultivations were conducted according to routine protocols as described in the material and methods part. 3 µl of diluted cell suspensions were spotted on CGXII agar plates with 2% (w/v) glucose. If not stated otherwise plates were incubated for 48 h at 30 °C.

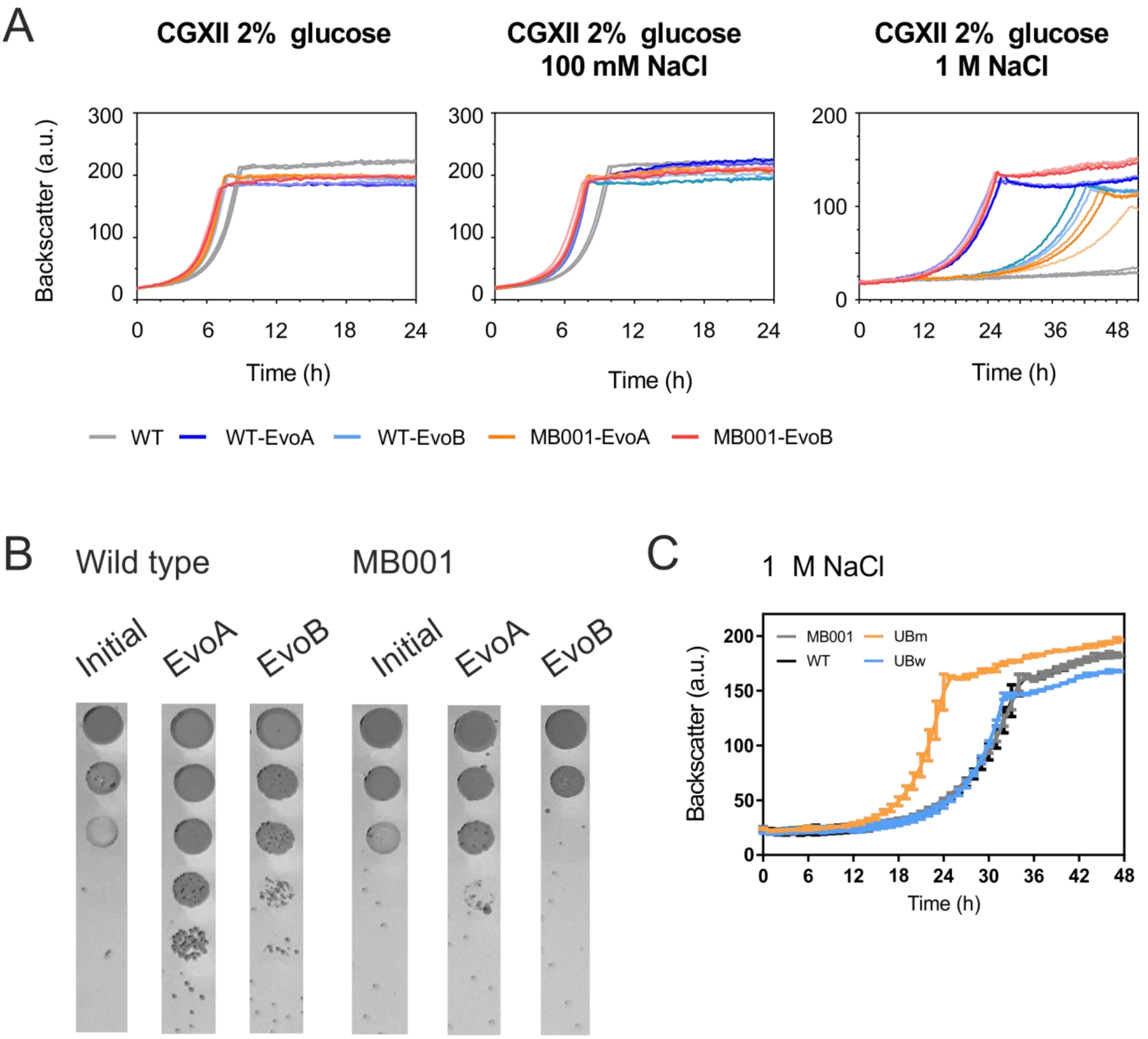

Figure S3: Osmotic stress tolerance of *C. glutamicum* wild type and evolved cell lines.
Osmotic stress tests were performed with *C. glutamicum* ATCC 13032 wild type and evolved cells (mixture after 630 generations) in liquid cultures (A) and agar spot tests (B). To test whether cells from this experiment (A, 1M NaCl) showed further adaptation to osmotic stress cells from the stationary phase of the 1 M NaCl experiment (from A) were spotted on agar plates with 1 M NaCl (B). Three technical replicates were tested (population mix, no single colonies) for each sample. Precultivations were conducted as described in the material and methods section. (C) The evolved strains, UBm and UBw, were tested for increased osmotic tolerance. Cultivations were conducted in CGXII media containing 2% (w/v) glucose and 1 M NaCl. Each graph represents three biological replicates (standard deviation included).

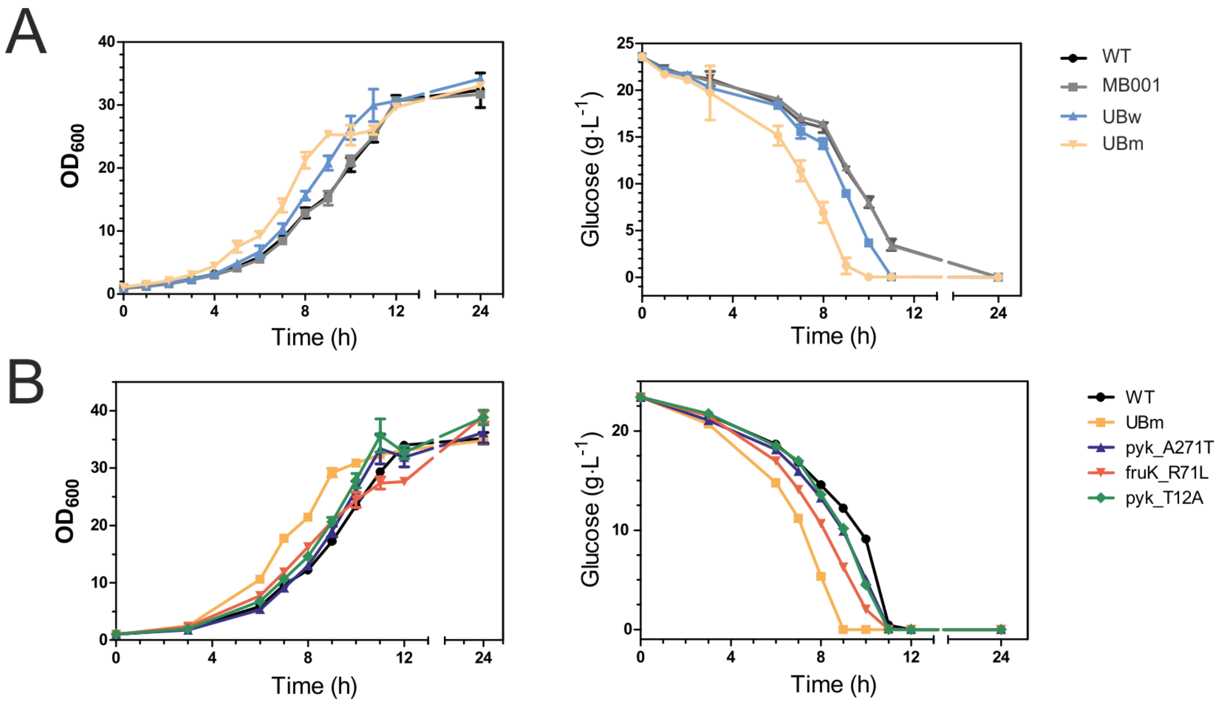

Figure S4: Growth and glucose consumption of initial, evolved and single-mutant strains.

A. Growth rates of evolved strains UBm and UBw were determined by shaking flask experiments. Glucose concentrations of supernatants were measured as described in the material and methods section and glucose consumption rates are listed in Table 3. Cells were cultivated in CGXII minimal media with 2% (w/v) glucose. Standard deviations are based on three biological replicates. **B** Glucose uptake rates of ATCC 13032 derived strains containing the mutations *pyk* A271T, *pyk* T12A and *fruK* R71L were determined and are also given in Table 3.

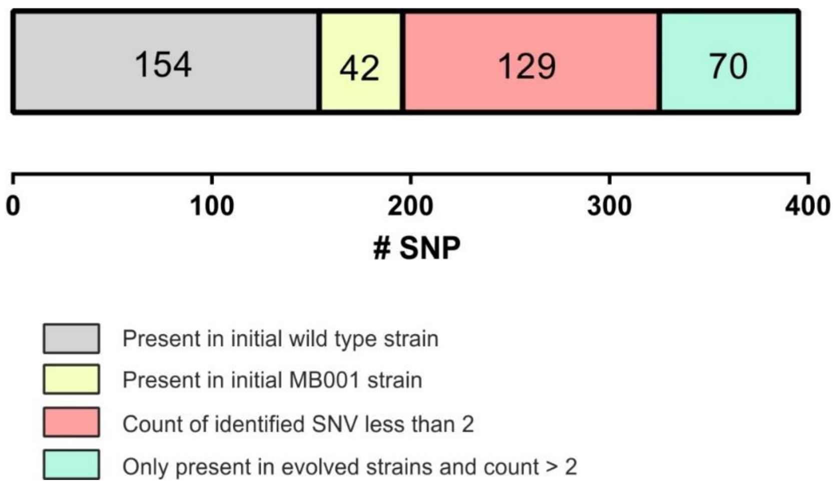

Figure S5: Evaluation of SNPs acquired from the ALE samples.

In sum 395 SNPs were identified by referring all evolved samples to the *C. glutamicum* ATCC 13032 genome sequence published in 2003[5]. 154 of them (grey) were also present within the initial wild type cells and are therefore excluded. Further 42 SNPs (yellow) were also found in the sequence of MB001 (and for this reason they are also excluded). 129 mutations (red) were counted less than two times and, thus are likely not responsible for the observed growth phenotypes. 70 SNPs (green) were only identified in evolved strains and were futher studied in detail.

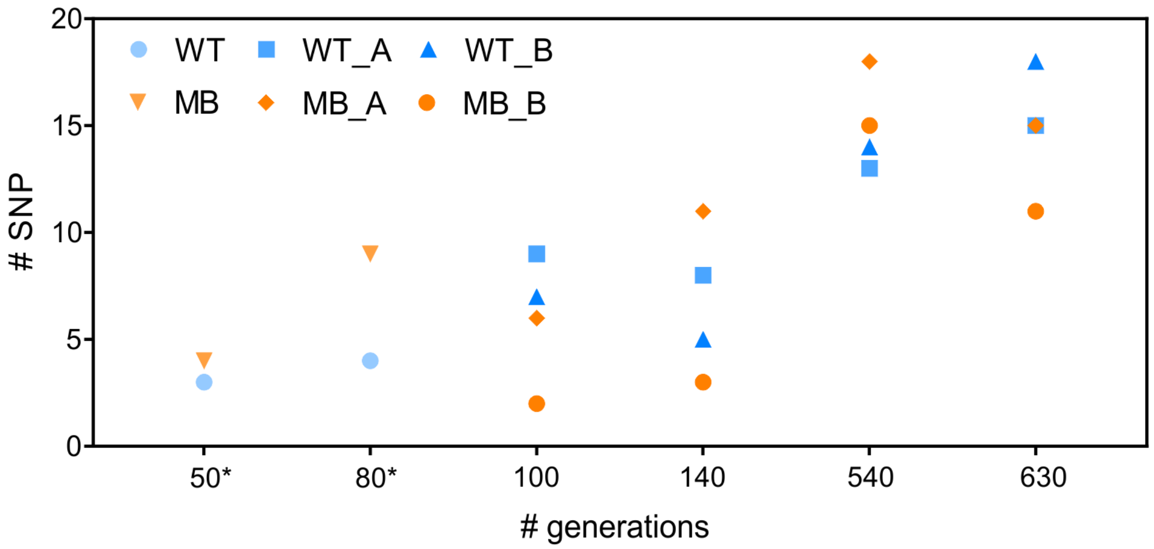

Figure S6: Distribution of the 70 relevant SNPs (presented in green in Figure S4).

Number of SNPs identified in ALE samples plotted for each time point. The amount of SNPs found in wild type strains (blue) and prophage-free strains (orange) increased over time. A and B represent two independent cell lines of the main experiment. Samples were taken after 100, 140, 540 and 630 generations. Samples marked with an asterisk represent samples from the second ALE experiment (only one cell line), which was conducted to verify the fitness leap after roughly 100 generations.

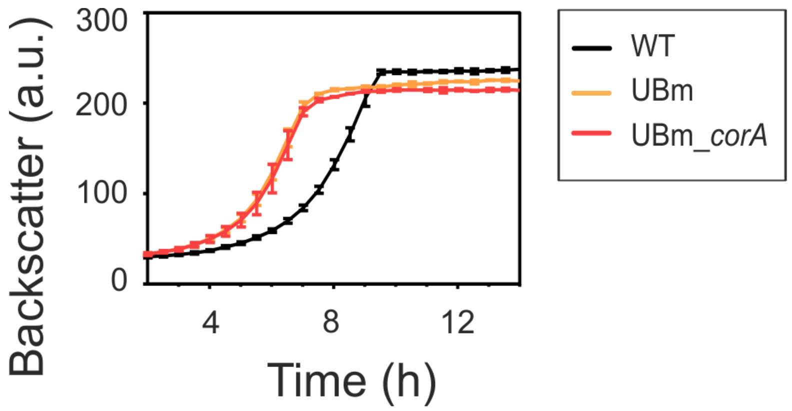

Figure S7: Restoration of the *corA* gene in the UBm strain.
After the mutated *corA* gene was replaced by the native *corA* in the UBm strain, it impact on the growth on glucose minimal media was investigated. As references, the wild type and the UBm stain were used. Data represent average values of three biological replicates.

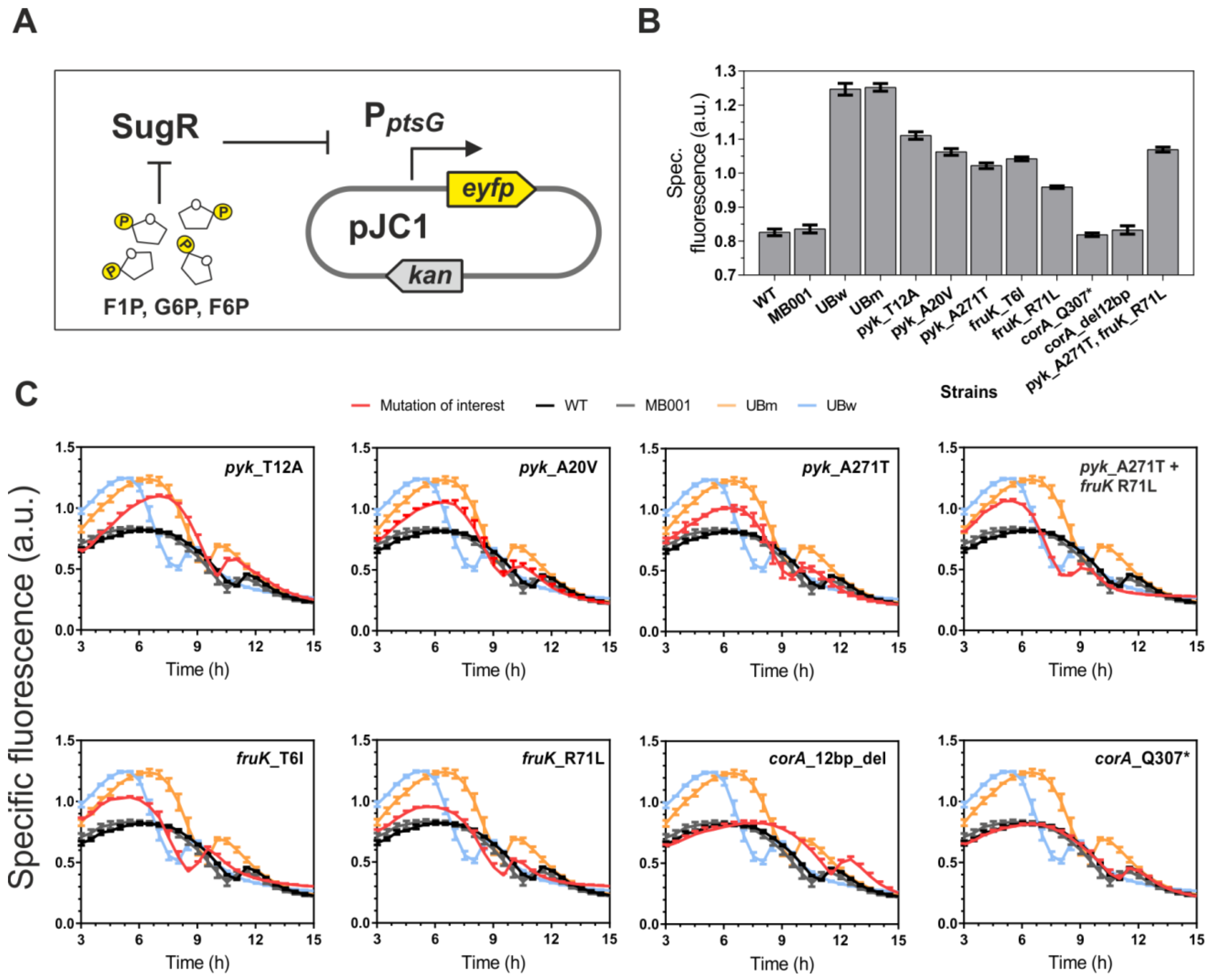

Figure S8: Impact of identified key mutations on *ptsG* expression.

A. A plasmid-based reporter was constructed to visualize expression of *ptsG* encoding enzyme 2 of the PTS system. The promoter region of *ptsG* (500 bp upstream) was fused to *eyfp* encoding the yellow fluorescent protein. The DeoR-type regulator SugR represses expression of *ptsG*, which can be relieved by hexose phosphates. Here, different effector molecules were reported in recent studies[6,7]. **B.** Fluorescence output of the *ptsG* reporter was measured in parental, evolved and single-mutant strains during cultivation in CGXII with 2% (w/v) glucose in microtiter plates. The maximum fluorescence (F) output is shown for each strain. Values were normalized to corresponding backscatter values (B) (F/ B =spec. fluorescence) **C.** Time course data of *ptsG* expression represented by the specific fluorescence of *ptsG* reporter. Corresponding growth curves are shown in Figure 5. The kink observed for the specific YFP fluorescence is a result of delayed chromophore maturation due to oxygen limitation in exponentially growing cells.

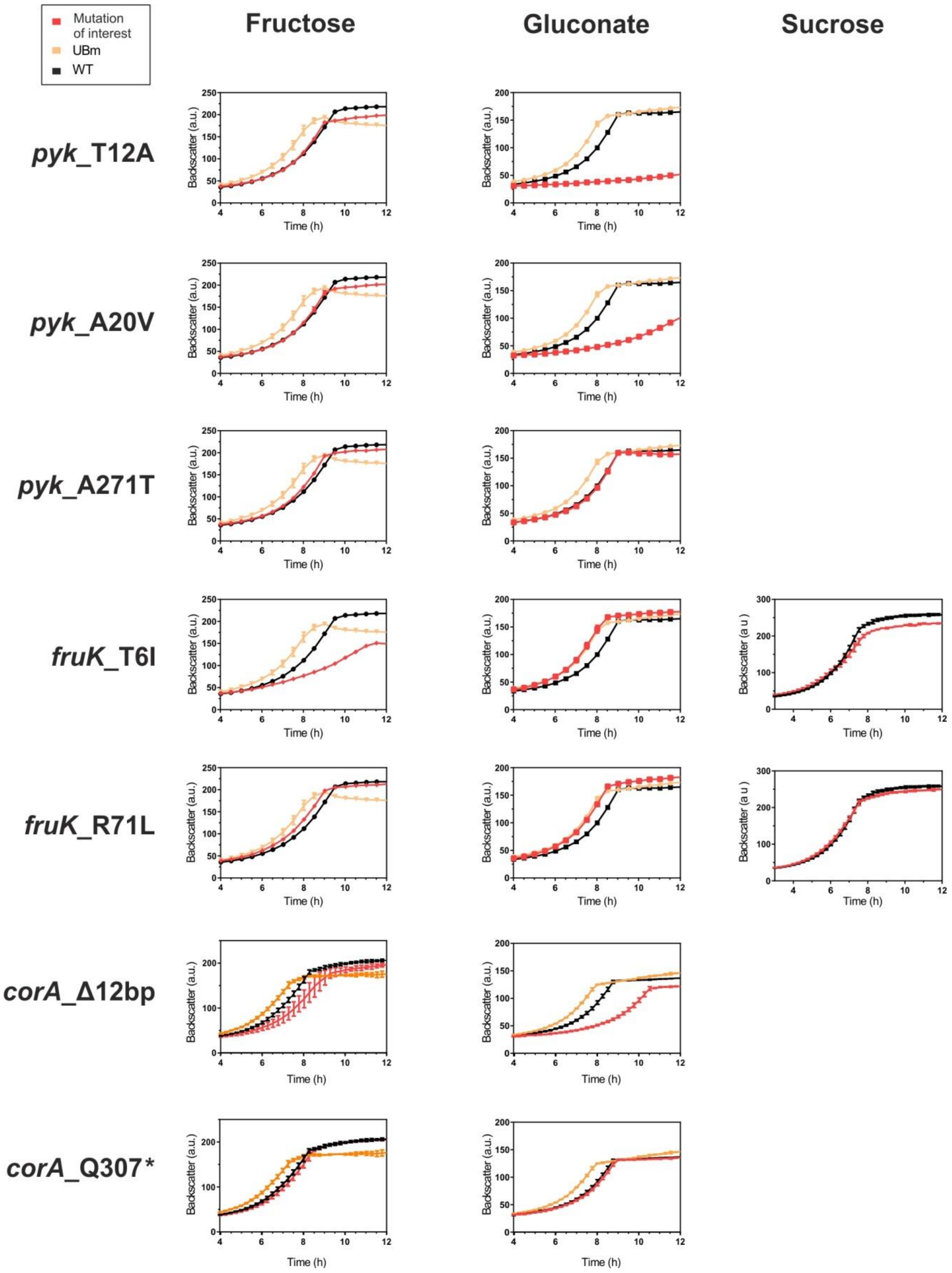

Figure S9: Growth experiments of mutant strains with different carbon sources.

To investigate the impacts of single mutations on the utilization of fructose, gluconate and sucrose mutated strains were cultivated in CGXII with 2% (w/v) fructose (left), 2% (w/v) gluconate (middle) or 2% sucrose (right) in microtiter plates. The wild type and the UBm strain were used as references. The data represent average values of three biological replicates including standard deviation except for sucrose where at least two biological replicates were used.

References

1 Kinoshita, S., Udaka, S. & Shimono, M. Studies on the amino acid fermentation. Part 1. Production of L-glutamic acid by various microorganisms. *J Gen Appl Microbiol* **50**, 331-343 (1957).

2 Baumgart, M. *et al.* Construction of a prophage-free variant of *Corynebacterium glutamicum* ATCC 13032 for use as a platform strain for basic research and industrial biotechnology. *Applied and environmental microbiology* **79**, 6006-6015, doi:10.1128/AEM.01634-13 (2013).

3 Schäfer, A. *et al.* Small mobilizable multi-purpose cloning vectors derived from the *Escherichia coli plasmids* pK18 and pK19: selection of defined deletions in the chromosome of *Corynebacterium glutamicum*. *Gene* **145**, 69-73 (1994).

4 Cremer, J., Eggeling, L. & Sahm, H. Cloning the Dapa Dapb Cluster of the Lysine-Secreting Bacterium *Corynebacterium-Glutamicum*. *Mol Gen Genet* **220**, 478-480, doi:Doi 10.1007/Bf00391757 (1990).

5 Kalinowski, J. *et al.* The complete *Corynebacterium glutamicum* ATCC 13032 genome sequence and its impact on the production of L-aspartate-derived amino acids and vitamins. *Journal of biotechnology* **104**, 5-25 (2003).

6 Engels, V. & Wendisch, V. F. The DeoR-type regulator SugR represses expression of *ptsG* in *Corynebacterium glutamicum*. *J Bacteriol* **189**, 2955-2966, doi:JB.01596-06 [pii]

10.1128/JB.01596-06 (2007).

7 Gaigalat, L. *et al.* The DeoR-type transcriptional regulator SugR acts as a repressor for genes encoding the phosphoenolpyruvate:sugar phosphotransferase system (PTS) in *Corynebacterium glutamicum*. *BMC molecular biology* **8**, 104, doi:10.1186/1471-2199-8-104 (2007).
